# Specific Assay Protocols for Porcine Single-Eye Retinal Pigment Epithelium Concerning Oxidative Stress and Inflammation

**DOI:** 10.3390/ijms26178434

**Published:** 2025-08-29

**Authors:** Philipp Dörschmann, Marie Prinz, Greta Schmitkall, Johann Roider, Alexa Klettner

**Affiliations:** Department of Ophthalmology, University Medical Center, Kiel University, Arnold-Heller-Str. 3, Haus B2, 24105 Kiel, Germanyalexa.klettner@uksh.de (A.K.)

**Keywords:** 3R principle, best practice protocol, primary cell culture, retinal pigment epithelium, oxidative stress, inflammation, single-eye cultures, age-related macular degeneration treatment

## Abstract

The retinal pigment epithelium (RPE) is strongly involved in the pathogenesis of several retinal diseases, such as age-related macular degeneration (AMD). RPE models addressing specific pathological pathways are of high importance for understanding cellular pathomechanisms and pre-clinical screening of potential new therapeutics. The goal of this study is to establish standard operation protocols for single-eye porcine RPE preparation for AMD-relevant models of oxidative stress (RPE-Ox) and inflammation (RPE-Inf). Porcine primary RPE were prepared from one eye and seeded into one well of 12-well plates or, for polar differentiation, in transwell inserts. Different coatings (Poly-ᴅ-Lysine and laminin) and serum content of media (10%, 5%, and 1%) were tested to determine optimal culture parameters. For RPE-Ox, cells were treated with NaIO_3_, CoCl_2_, or erastin; cell viability (thiazolyl blue tetrazolium bromide, MTT), and gene expression (RT-qPCR) were determined. For RPE-Inf, cells were treated with lipopolysaccharide (LPS), polyinosinic/polycytidylic acid (Poly I:C), or tumor necrosis factor alpha (TNF-α); cell viability (MTT), cytokine secretion (ELISA), and gene expression (RT-qPCR) were determined. For transwell plates in RPE-Inf, cell viability (MTT), polar cytokine secretion (ELISA), gene expression (RT-qPCR), and transepithelial electrical resistance (TEER) for barrier assessment were conducted. For RPE-Ox, effective LD_50_ could be achieved by using 24 h stimulation with 25 µm erastin, seven days after preparation in 5% serum cultures, without coating. For gene expression assessment, the use of Poly-ᴅ-Lysine is recommended. For RPE-Inf, three days of LPS stimulation (1 µg/mL) showed effective cytokine activation with 5% serum on uncoated 12-well plates. Transwell plates are not recommended for cytokine secretion assessment. It can be used for cell barrier assays in which LPS also showed effective cell barrier decrease and gene expression assays. Two specific best practice protocols for the use of porcine single-eye cultures in AMD research concerning oxidative stress and inflammation with optimized parameters were established and are provided.

## 1. Introduction

The investigation of diseases and the development of new therapeutics needs appropriate pre-clinical models. An important cell in the development of several retinal diseases, such as age-related macular degeneration (AMD), is the retinal pigment epithelium (RPE). The RPE is located between the choroid and the photoreceptors, where it constitutes the outer blood–retinal barrier. The RPE is vital for visual function, as it maintains the photoreceptors, e.g., by recycling the visual pigment, discarding their waste, or by providing them with nutrients [1]. Several models of RPE culture exist, such as the ARPE-19 cell line, iPSC-RPE (induced pluripotent stem cell RPE), or mixed porcine RPE cultures, each having its advantages and limitations [2]. We recently introduced porcine single-eye RPE cell culture, establishing a best practice protocol for highly differentiated RPE cells [3], which was recently optimized for studies of barrier and polarity [4]. This cell culture has several advantages. It is derived from porcine eyes, which show strong homology to human eyes in anatomy, retinal structure, and gene expression [5,6,7]. These eyes are slaughterhouse waste material and therefore can be obtained without the need of killing animals solely for scientific purposes. They can be cultured with a relatively easy protocol for extended periods of time and reach a high degree of differentiation [3]. In addition, because each culture is derived from one eye, the cells are genetically homogeneous within one culture and genetically heterogeneous between the cultures, therefore resembling the in vivo situation more closely than cell lines, inbred strains, or RPE mixed cultures. A drawback of this model is the higher variability in the results, due to the different genetic backgrounds of the cells [3]. However, this challenge can be met by a higher sample size and appropriate statistical analysis [8]. Furthermore, the higher variability potentially makes any significant results biologically more relevant, which is a clear asset of the model. Taken together, porcine single-eye RPE cell cultures are a biologically relevant, economic, and sustainable model.

**Figure 1:**
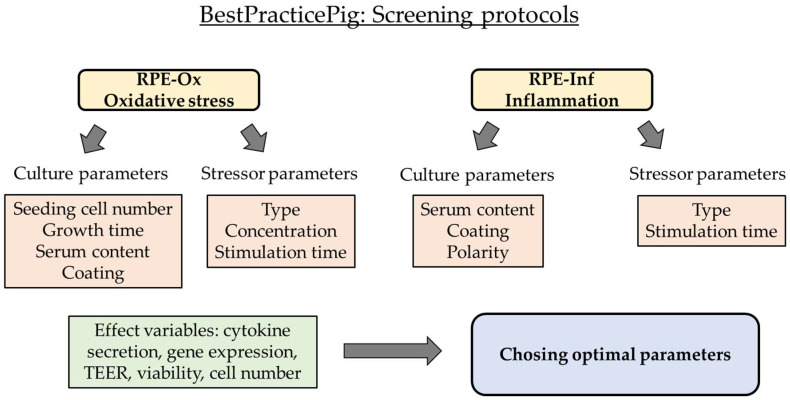
Flowchart of experimental schedule. The goal of this study is to establish two specific model systems with single-eye cultures from porcine retinal pigment epithelium (RPE). Cells from one pig eye are seeded into one well of a 12-well plate or transwell insert, generating genetically homogenous cell systems. For the oxidative stress model (RPE-Ox) and the inflammation model (RPE-Inf), culture parameters and different stressor agents are optimized for reproducible effects. Measuring parameters are listed (effect variables). TEER = transepithelial electrical resistance.

Relevant pathomechanisms in the RPE in AMD development are oxidative stress and inflammation [9,10,11]. In this study, based on our previously published protocol, we aimed to establish a protocol for optimized and reproducible testing of oxidative stress and inflammatory activation of RPE cells (please refer to Figure 1 for the project overview). For oxidative stress, we tested several commonly used oxidative stressors (H_2_O_2_, CoCl_2_, and erastin [12,13,14]) to evaluate the best stressor, the optimal concentration, and the appropriate incubation time. In addition, we investigated the influence of cell culture parameters such as serum content and coating. For pro-inflammatory activation, we tested lipopolysaccharide (LPS) and polyinosinic/polycytidylic acid (Poly I:C, PIC), as both have been shown to induce a strong pro-inflammatory activation in mixed porcine RPE cultures [15], elucidating the best concentrations and time points. Again, we investigated the influence of coating and serum content. In addition, we compared polarized and non-polarized cultures.

In our study, we could identify best practice protocols for oxidative stress and inflammation testing in porcine single-eye RPE cells. For oxidative stress, we recommend seven days of cultivation followed by 24 h of stimulation with 25 µm erastin in uncoated 5% serum cultures. For pro-inflammatory activation, we recommend three days of 1 µg/mL LPS stimulation in uncoated 5% serum cultures.

## 2. Results

### 2.1. Establishing an Oxidative Stress Model with Single-Eye Cultures (RPE-Ox)

#### 2.1.1. Optimal Conditions for Cell Survival Reduction (50%)

To establish effective cell survival assays with single-eye cultures, different parameters, including cultivation time, coatings, and serum content, as well as different stressor agents with different concentrations and stimulation times, were tested. Cell viability was assessed with thiazolyl blue tetrazolium bromide (MTT assay). Preliminary tests with single-eye RPE concerning optimal MTT incubation time (15 to 60 min) to achieve linear range in absorption measurement were performed. MTT incubation time of 15 min was optimal. Also, a suitable cell seeding number was determined for experiments (75,000 to 150,000 cells/mL), of which 100,000 cells/mL was the most suitable and reproducible.

For all experiments, cell viability of the untreated control (without stress agents) was set to 100%, and all stimulated conditions were set in relation (in %). As stressors, 250 µM H_2_O_2_, 500 µM H_2_O_2_, 20 µM erastin, 25 µM erastin, 10 mM NaIO_3_, 20 mM NaIO_3_, 250 µM CoCl_2_, and 500 µM CoCl_2_ were used for oxidative stress induction. Choices for stressors and concentrations are based on preliminary tests, experiences with mixed RPE cultures, and literature findings [14,16]. A reduction in viability of approximately 50% was aimed for.

**Figure 2:**
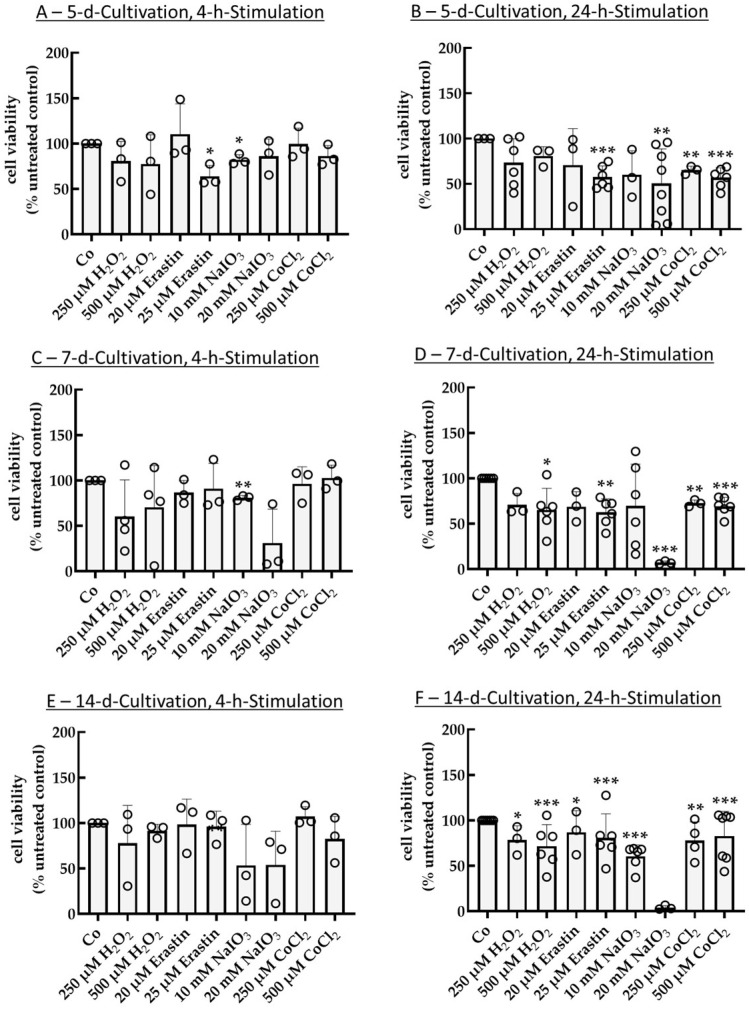
Oxidative stress insult in single-eye cultures, initial tests. Primary porcine retinal pigment epithelium single-eye cultures were treated with 250 µM H_2_O_2_, 500 µM H_2_O_2_, 20 µM erastin, 25 µM erastin, 10 mM NaIO_3_, 20 mM NaIO_3_, 250 µM CoCl_2_, or 500 µM CoCl_2_ for 4 h (**A**,**C**,**E**) or 24 h (**B**,**D**,**F**) after a cultivation time of 5 d (**A**,**B**), 7 d (**C**,**D**), or 14 d (**E**,**F**) on uncoated 12-well plates using 10% serum. Tetrazolium bromide assay (MTT) was used to determine cell viability. Absorption of an untreated control was set to 100%, and other samples were calculated in relation. Data were parametric. Significances were calculated with a one-sample *t*-test. * *p* < 0.05, ** *p* < 0.01, *** *p* < 0.001, *n* = 3–8. Co = control, circles = individual data points.

First, different stimulation (4 h and 24 h) and cultivation times (5 d, 7 d, 14 d) were tested without coating and with 10% serum content of media. After 5 d of cultivation and 4 h stimulation time (Figure 2A), 25 µm erastin and 10 mM NaIO_3_ reduced cell viability to 64.06% ± 11.70% (*p* = 0.030) and 82.10% ± 5.62% (*p* = 0.030), respectively. Neither of the conditions reached LD_50_, and the other tested conditions showed no significant effects. After 5 d of cultivation and 24 h stimulation time (Figure 2B), stress insults displayed more pronounced effects. Here, 25 µM erastin and 20 mM NaIO_3_ reduced viability to 57.44% ± 12.44% (*p* < 0.001) and 50.42% ± 38.16% (*p* = 0.030). Also, 250 µM and 500 µM CoCl_2_ reduced viability to 65.12% ± 4.38% (*p* = 0.005) and 56.73% ± 11.06% (*p* < 0.001), respectively. NaIO_3_ showed an ideal LD_50_ value; however, variation was high. After 7 d of cultivation and 4 h of stimulation (Figure 2C), only NaIO_3_ showed a significant reduction in cell viability to 81.00% ± 2.65% (*p* = 0.006). No other conditions reached significance, although 20 mM NaIO_3_ displayed a strong, yet insignificant effect (31.07% ± 37.29%). After 7 d of cultivation and 24 h stimulation (Figure 2D), 500 µM H_2_O_2_, 25 µM erastin, and 20 mM NaIO_3_ reduced cell viability to 65.11% ± 23.96% (*p* = 0.020), 62.71% ± 14.75% (*p* = 0.002), or 7.18% ± 1.70% (*p* < 0.001), while 250 µM and 500 µM CoCl_2_ showed values of 72.48% ± 3.64% (*p* = 0.006) and 70.27% ± 9.50% (*p* < 0.001), respectively. After 14 d of cultivation and 4 h of stimulation (Figure 2E), no condition showed significant results. Of note, 10 mM NaIO_3_ (53.20% ± 45.30%) and 20 mM NaIO_3_ (53.90% ± 36.94%) nearly reached LD_50_ but reached no significance due to high variation. After 14 d of cultivation and 24 h stimulation (Figure 2F), besides 20 mM NaIO_3_, which killed nearly all cells (4.01% ± 2.29%) without reaching significance, all conditions showed significant effects (250 µM H_2_O_2_: 78.37% ± 15.63% (*p* = 0.010); 500 µM H_2_O_2_: 71.52% ± 23.81% (*p* < 0.001); 20 µM erastin: 86.94% ± 23.74% (*p* = 0.020); 25 µM erastin: 80.69% ± 26.54% (*p* < 0.001); 10 mM NaIO_3_: 60.41% ± 12.59% (*p* < 0.001); 250 µM CoCl_2_: 77.88% ± 20.47% (*p* = 0.005); 500 µM CoCl_2_: 82.74% ± 27.11% (*p* < 0.001)). Here, no condition reached LD_5,_ but they displayed less variability. Taken together, 25 µm erastin, 20 mM NaIO_3_, and 500 µM CoCl_2_ with 5 d of cultivation and 24 h stimulation time were efficient (reaching nearly 50% viability). Conversely, 4 h of stimulation and 14 d of cultivation time were clearly not effective and were excluded from further assays. Serum level tests followed.

**Figure 3:**
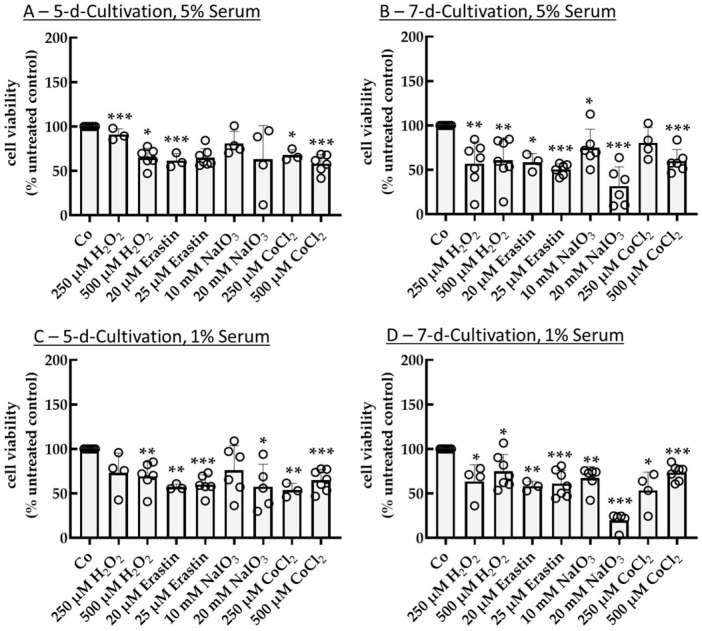
Oxidative stress insult in single-eye cultures, serum tests. Primary porcine retinal pigment epithelium single-eye cultures were treated with 250 µM H_2_O_2_, 500 µM H_2_O_2_, 20 µM erastin, 25 µM erastin, 10 mM NaIO_3_, 20 mM NaIO_3_, 250 µM CoCl_2_, or 500 µM CoCl_2_ for 24 h after a cultivation time of 5 d (**A**,**C**) or 7 d (**B**,**D**) on uncoated 12-well plates using 5% (**A**,**B**) or 1% (**C**,**D**) serum. Tetrazolium bromide assay (MTT) was used to determine cell viability. Absorption of an untreated control was set to 100%, and other samples were calculated in relation. Data were parametric. Significances were calculated with a one-sample *t*-test. * *p* < 0.05, ** *p* < 0.01, *** *p* < 0.001, *n* = 3–7. Co = control, circles = individual data points.

For further tests, serum level was reduced to 5% and 1%, uncoated plates were used, and cultivation times of 5 d and 7 d with 24 h stimulation were tested. Using 5% serum for 5 d of cultivation (Figure 3A), a significant reduction in cell viability was achieved by 500 µM H_2_O_2_, 20 µM erastin, and 25 µM erastin with 64.93% ± 10.66% (*p* < 0.001), 61.60% ± 8.00% (*p* = 0.010), or 64.97% ± 10.06% (*p* < 0.001), as well as 250 µM and 500 µM CoCl_2_ with 67.69% ± 6.23% (*p* = 0.010) and 58.27% ± 10.24% (*p* < 0.001), respectively. Using 5% serum for 7 d of cultivation (Figure 3B), an effective reduction in cell survival by 250 µM H_2_O_2_ (56.82% ± 24.76% (*p* = 0.004)), 500 µM H_2_O_2_ (60.94% ± 24.96% (*p* = 0.006)), 20 µM erastin (58.43% ± 9.96% (*p* = 0.020)), 25 µM erastin (50.40% ± 6.56% (*p* < 0.001)), 10 mM NaIO_3_ (74.93% ± 20.88% (*p* = 0.030)), 20 mM NaIO_3_ (31.74% ± 21.57% (*p* < 0.001)), as well as 500 µM CoCl_2_ (59.91% ± 13.05% (*p* < 0.001)) was shown. Here, 25 µM erastin displayed a highly reproducible LD_50_. Regarding 1% serum and 5 d cultivation (Figure 3C), 500 µM H_2_O_2_, 20 µM erastin, and 25 µM erastin reduced viability to 69.36% ± 15.92% (*p* = 0.005), 57.22% ± 3.17% (*p* = 0.002), and 59.81% ± 11.06% (*p* < 0.001), while 20 mM NaIO_3_, 250 µM CoCl_2_, and 500 µM CoCl_2_ significantly reduced cell viability to 57.46% ± 25.34% (*p* = 0.020), 53.60% ± 7.80% (*p* = 0.009), and 64.92% ± 12.04% (*p* < 0.001), respectively. None of the conditions reached values equal to or below 50%. Finally, regarding 1% serum and 7 d cultivation (Figure 3D), all conditions exhibited significant cell death (250 µM H_2_O_2_: 63.54% ± 18.67% (*p* = 0.030); 500 µM H_2_O_2_: 74.93% ± 18.93% (*p* = 0.010); 20 µM erastin: 58.21% ± 5.32% (*p* = 0.005); 25 µM erastin: 61.11% ± 14.82% (*p* < 0.001); 10 mM NaIO_3_: 67.25% ± 12.89% (*p* = 0.002); 20 mM NaIO_3_: 19.50% ± 9.29% (*p* < 0.001); 250 µM CoCl_2_: 53.25% ± 20.64% (*p* = 0.020); 500 µM CoCl_2_: 73.38% ± 8.63% (*p* < 0.001)). Taken together, 25 µM erastin showed the most effective oxidative stress result with 5% serum, 24 h stimulation, and 7 d cultivation time.

**Figure 4:**
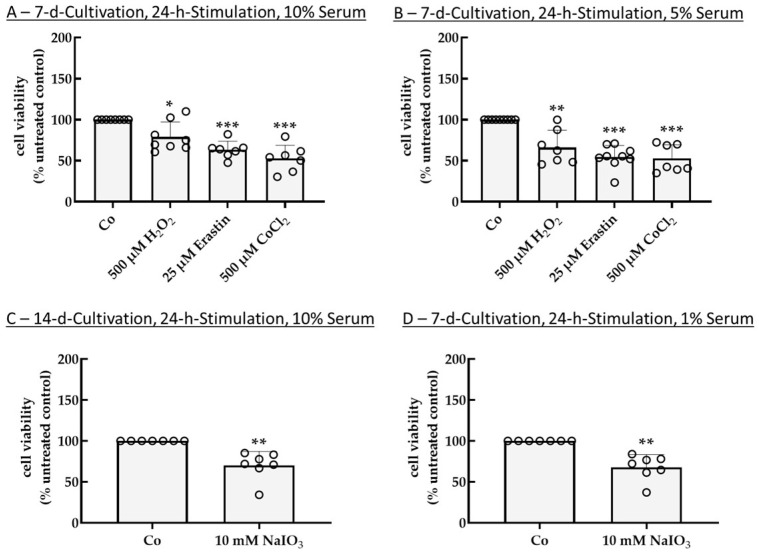
Oxidative stress insult in single-eye cultures, coating tests. Primary porcine retinal pigment epithelium single-eye cultures were treated with 500 µM H_2_O_2_, 25 µM erastin, 500 µM CoCl_2_ (**A**,**B**), or 10 mM NaIO_3_ (**C**,**D**) for 24 h after a cultivation time of 7 d (**A**,**C**,**D**) or 14 d (**C**) on 12-well plates coated with Poly-ᴅ-Lysine using 10% (**A**,**C**), 5% (**B**), or 1% (**D**) serum. Tetrazolium bromide assay (MTT) was used to determine cell viability. Absorption of an untreated control was set to 100%, and other samples were calculated in relation. Data were parametric. Significances were calculated with a one-sample *t*-test. * *p* < 0.05, ** *p* < 0.01, *** *p* < 0.001, *n* = 7–9. Co = control, circles = individual data points.

As use of Poly-ᴅ-Lysine (PDL) proved to be beneficial in single-eye RPE cultivation [3], tests were repeated with PDL coating and the most promising parameters of the former results. Regarding 7 d cultivation, 24 h stimulation time, and 10% serum (Figure 4A), all tested conditions, namely 500 µM H_2_O_2_, 25 µm erastin, and 500 µM CoCl_2_, reduced cell viability to 79.07% ± 18.01% (*p* = 0.010), 63.31% ± 10.44% (*p* < 0.001), and 52.67% ± 16.17% (*p* < 0.001), respectively. Under these conditions, CoCl_2_ showed the most effective stress insult and was more effective compared to the results with no coating (70.27% ± 9.50%), whereas erastin was not influenced by coating and showed similar results as with no coating (62.71% ± 14.75%). Conversely, H_2_O_2_ was more effective without coating (65.11% ± 23.96%). Regarding 7 d cultivation, 24 h stimulation time, and 5% serum (Figure 4B), again, all tested conditions reduced cell viability (500 µM H_2_O_2_: 66.22% ± 20.87% (*p* = 0.005); 25 µm erastin: 54.44% ± 14.07% (*p* < 0.001); 500 µM CoCl_2_: 52.60% ± 16.76% (*p* < 0.001)), with erastin and CoCl_2_ being similarly effective. All three stressor agents were slightly less efficient compared to no-coating conditions (H_2_O_2_: 60.94% ± 24.96%; erastin: 50.40% ± 6.56%; CoCl_2_: 59.91% ± 13.05%). By using 10 mM NaIO_3_, cell viability for cells coated on PDL could be successfully reduced with 14 d cultivation, 24 h stimulation, and 10% serum (Figure 4C) as well as 7 d cultivation, 24 h stimulation, and 1% serum (Figure 4D). Here, viability could be reduced to 69.99% ± 17.13% (*p* = 0.004) (which was higher compared to no coating with 60.41% ± 12.59%) and 67.68% ± 15.57% (*p* = 0.002) (which was similar compared to no coating with 67.25% ± 12.89%), respectively. These results indicate that PDL coating is not necessary for oxidative stress survival models.

**Figure 5:**
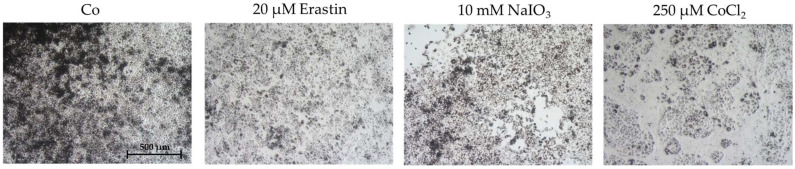
Exemplary photos after oxidative stress insult. After 24 h stimulation of single-eye retinal pigment epithelium in 12-well plates (7 days of cultivation, using 5% serum without coating), cells were analyzed via light microscopy for cell morphology. Exemplary photos (5× objective) for untreated control (Co), 25 µM erastin, 10 mM NaIO_3_, and 500 µM CoCl_2_ are shown. Scale bar = 500 µm.

Of note, cells were also investigated for cell morphology after stress insults. None of the tested compounds drastically changed cell morphology besides a reduction in cell number and small mesenchymal transitions in the cell layer gaps. Exemplary photos are shown in Figure 5.

#### 2.1.2. Expression of Oxidative Stress-Related Genes

To assess oxidative stress-related gene expression, primary porcine RPE of single-eye cultures were treated with the most promising parameters from Section 2.1.1. For this, 25 µM erastin, 500 µM CoCl_2_, or 10 mM NaIO_3_ were used for 24 h stimulation after a cultivation time of 7 d or 14 d on 12-well uncoated plates or plates coated with PDL using 10%, 5%, or 1% serum. RNA was isolated, and RT-qPCR was performed. *GUSB* was used as an endogenous control. Targets for oxidative stress were *CAT*, *GPX4*, *GSS*, *NFE2L2,* and *SOD1*. Data were evaluated with Thermo Fisher Connect and set in relation to the untreated control (Rq = 1.00) (Table 1).

Using 10 mM NaIO_3_ after a cultivation time of 7 d with 1% serum exhibited a significant decrease in *CAT* (Rq = 0.11, *p* = 0.023). The expression of the other four genes also decreased; however, significance was not reached. With the same conditions but using PDL as a coating (7 d cultivation, 1% serum), all genes were reduced by 10 mM NaIO_3_, with *CAT* (Rq = 0.12, *p* < 0.001), *GPX4* (Rq = 0.23, *p* = 0.045), and *NFE2L2* (Rq = 0.22, *p* = 0.021) reaching significance and *GSS* and *SOD1* not. By using 5% serum with a 7d cultivation time, the only significant effect was seen with 25 µm erastin on PDL-coated wells; here, *SOD1* was significantly increased (Rq = 3.04, *p* = 0.011). By using 10% serum and a 7d cultivation time, only 25 µm erastin on PDL-coated wells displayed a significant effect, increasing *NFE2L2* expression (Rq = 2.84, *p* = 0.036). By using 10 mM NaIO_3_ after a cultivation time of 14 d together with 10% serum, a significant reduction in *CAT* was found (Rq = 0.21, *p* = 0.024), while using PDL with these conditions, in addition to *CAT* (Rq = 0.10, *p* = 0.003), *GPX4* (Rq = 0.05, *p* = 0.015) was significantly reduced too. Taken together, NaIO_3_ reduced *CAT* expression under several conditions. Also, when PDL is used, erastin significantly increased gene expression for *SOD1* and *NFE2L2*. While we do not recommend using PDL for the cell death assay, it should be used if gene expression is an objective of this study.

### 2.2. Establishing an Inflammation Model with Single-Eye Cultures (RPE-Inf)

#### 2.2.1. Cell Viability

**Figure 6:**
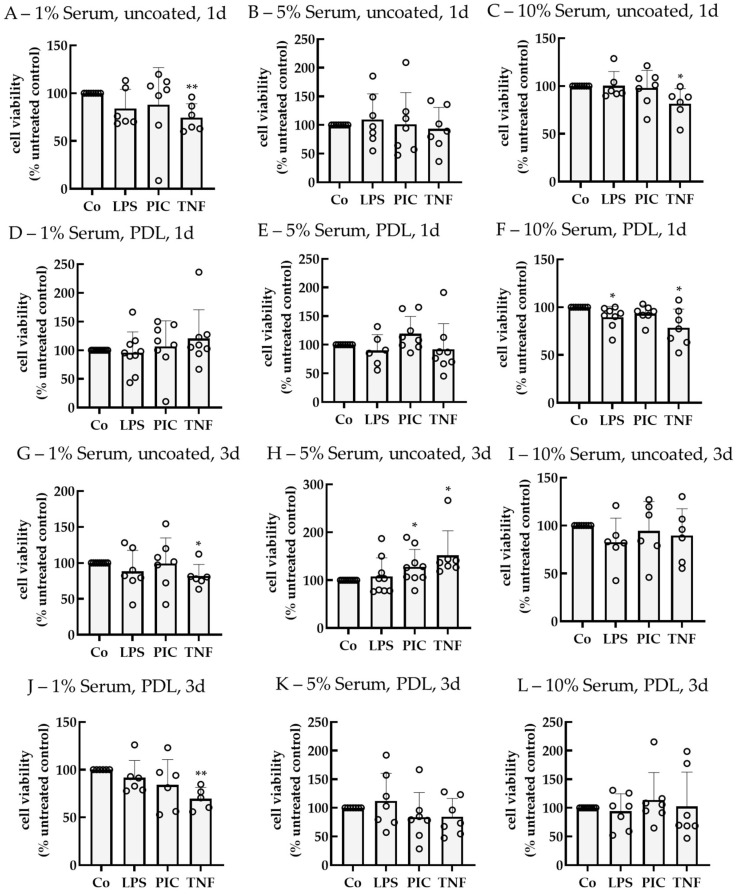
Cell viability of pro-inflammatorily activated porcine single-eye RPE cultures. Single-eye retinal pigment epithelium cells were stimulated on uncoated (**A**–**C**,**G**–**I**) or Poly-ᴅ-Lysine (PDL, (**D**–**F**,**J**–**L**)) coated 12-well plates with 1% (**A**,**D**,**G**,**J**), 5% (**B**,**E**,**H**,**K**), or 10% (**C**,**F**,**I**,**L**) serum-containing media, respectively. Cells were stimulated with 1 µg/mL lipopolysaccharide (LPS), 10 µg/mL polyinosinic/polycytidylic acid (Poly I:C, PIC), or 50 ng/mL tumor necrosis factor (TNF) for 1 (**A**–**F**) or 3 (**G**–**L**) d. Cell viability was determined with tetrazolium bromide assay (MTT). Data were parametric. Significances were calculated with a one-sample *t*-test against untreated controls (co), set to 100%. * *p* < 0.05, ** *p* < 0.01, *n* = 6–9. Co = control, circles = individual data points.

To test the influence of pro-inflammatory insult on single-eye RPE cell viability, cells were treated with LPS, PIC, or tumor necrosis factor alpha (TNF) after a cultivation time of 14–18 d. Cells were stimulated for 1 or 3 d. As cell culture parameters, PDL vs. uncoated wells and 1%, 5%, and 10% serum were tested. Cell viability was determined with the MTT assay and calculated as a % compared to the untreated control, which was set to 100% (Figure 6). The choice of stressors, tested concentrations, and stimulation times was based on previous studies investigating pro-inflammatory activation of porcine primary RPE in mixed cultures [15].

After 1 d of stimulation and using no coating, TNF reduced cell viability using 1% serum or 10% serum to 74.58% ± 14.53% (*p* = 0.008) and 81.65% ± 15.65% (*p* = 0.030), respectively. Using PDL and 10% serum, LPS reduced viability to 89.62% ± 11.37% (*p* = 0.040), while TNF reduced viability to 78.72% ± 19.38% (*p* = 0.030). After 3 d of stimulation, using 1% serum without coating, TNF reduced viability to 81.84% ± 16.23% (*p* = 0.040). In contrast, using 5% serum without coating, PIC and TNF increased cell viability to 128.42% ± 35.67% (*p* = 0.040) and 151.94% ± 51.26% (*p* = 0.040), respectively. Also, TNF decreased cell viability to 69.68% ± 11.73% (*p* = 0.040) if 1% serum and PDL were used. 5% serum without the use of coating seems to be suitable to investigate cytokine secretion without cell death.

**Figure 7:**
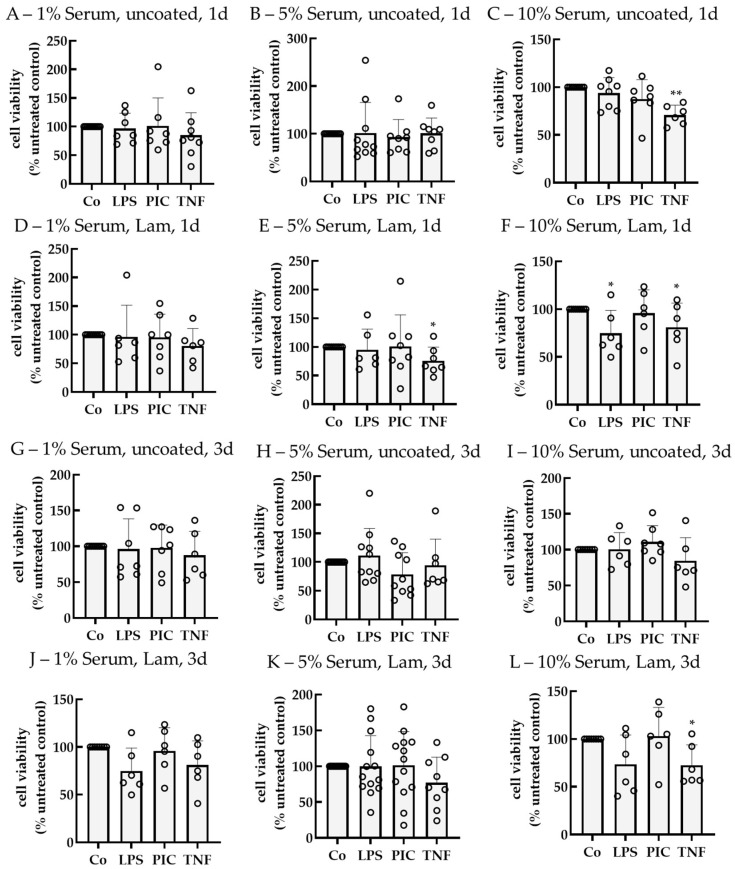
Cell viability of pro-inflammatorily activated polar porcine single-eye RPE cultures. Single-eye retinal pigment epithelium were stimulated on uncoated (**A**–**C**,**G**–**I**) or laminin (Lam, (**D**–**F**,**J**–**L**)) coated 12-well plates with transwell inserts with 1% (**A**,**D**,**G**,**J**), 5% (**B**,**E**,**H**,**K**), or 10% (**C**,**F**,**I**,**L**) serum-containing media. Cells were stimulated with 1 µg/mL lipopolysaccharide (LPS), 10 µg/mL polyinosinic/polycytidylic acid (Poly I:C, PIC), or 50 ng/mL tumor necrosis factor (TNF) for 1 (**A**–**F**) or 3 (**G**–**L**) d. Cell viability was determined with tetrazolium bromide assay (MTT). Data were parametric. Significances were calculated with a one-sample *t*-test against untreated controls (co), set to 100%. * *p* < 0.05, ** *p* < 0.01, *n* = 6–10. Co = control, circles = individual data points.

Experiments were repeated with polar single-eye RPE on transwell inserts using laminin (Lam) instead of PDL as a coating (Figure 7). After 1 d of stimulation, using 10% serum without coating, TNF reduced cell viability to 71.00% ± 10.37% (*p* = 0.001). Using 5% serum with Lam coating, TNF reduced cell viability to 75.81% ± 23.97% (*p* = 0.037), while using 10% serum with Lam, LPS, and TNF reduced viability to 76.87% ± 20.39% (*p* = 0.015) and 71.50% ± 22.84% (*p* = 0.028), respectively. After 3 d of stimulation, only TNF on Lam in 10% serum reduced cell viability (72.62% ± 21.27% (*p* = 0.030)). Taken together, using LPS and 5% serum without coating are appropriate conditions for stable viability and non-toxic effects when investigating pro-inflammatory effects.

**Figure 8:**
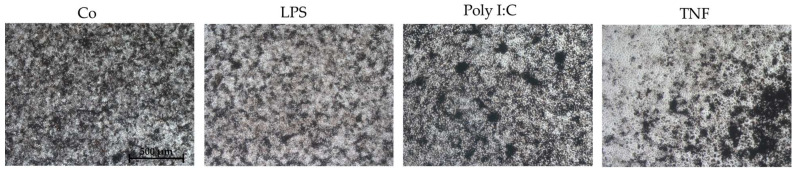
Exemplary photos after inflammation stress insult. After 3 days of stimulation of single-eye retinal pigment epithelium in 12-well plates (using 5% serum without coating), cells were analyzed via light microscopy for cell morphology. Exemplary photos (5× objective) for untreated control (Co), 1 µg/mL lipopolysaccharide (LPS), 10 µg/mL polyinosinic/polycytidylic acid (Poly I:C), and 50 ng/mL tumor necrosis factor (TNF) are shown. Scale bar = 500 µm.

Cells were also investigated for cell morphology after inflammatory stress insults. None of the tested compounds relevantly changed cell morphology. Exemplary photos are shown in Figure 8.

#### 2.2.2. Cytokine Secretion—Non-Polar

**Figure 9:**
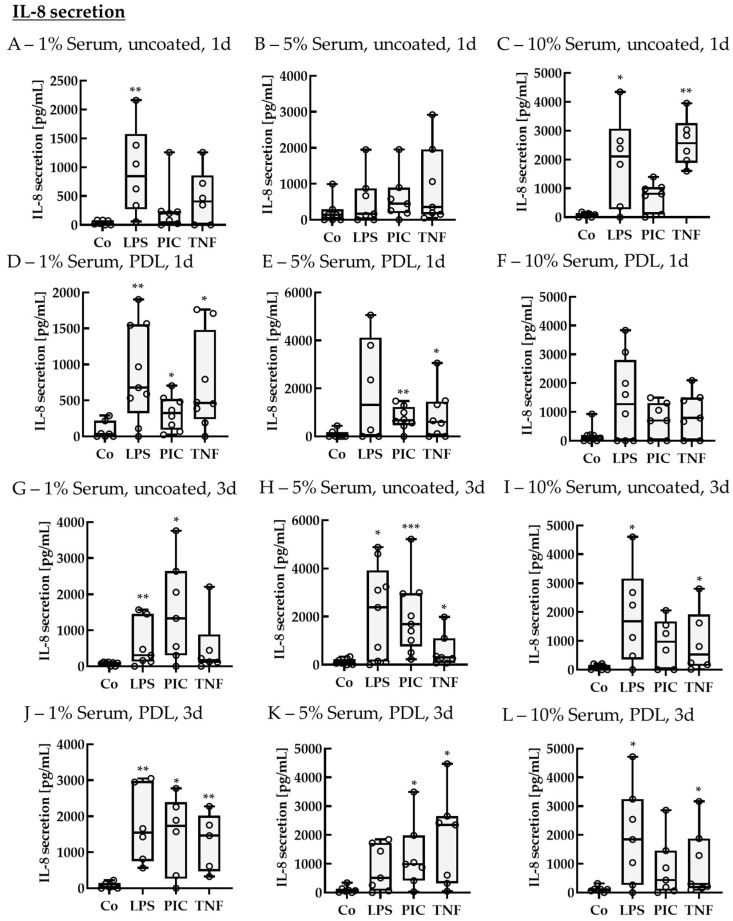
Interleukin 8 secretion of pro-inflammatorily activated porcine single-eye RPE cultures. Single-eye retinal pigment epithelium cultures were stimulated on uncoated (**A**–**C**,**G**–**I**) or Poly-ᴅ-Lysine (PDL, (**D**–**F**,**J**–**L**)) coated 12-well plates with 1% (**A**,**D**,**G**,**J**), 5% (**B**,**E**,**H**,**K**), or 10% (**C**,**F**,**I**,**L**) serum-containing media. Cells were stimulated with 1 µg/mL lipopolysaccharide (LPS), 10 µg/mL polyinosinic/polycytidylic acid (Poly I:C, PIC), or 50 ng/mL tumor necrosis factor (TNF) for 1 (**A**–**F**) or 3 (**G**–**L**) d. Supernatants were collected and applied in ELISA to detect secreted interleukin 8 (IL-8). Data were not parametric. Significances were calculated with Kruskal–Wallis and Mann–Whitney tests against untreated control (Co). * *p* < 0.05, ** *p* < 0.01, *** *p* < 0.01, *n* = 6–9. Circles = individual data points.

To test the influence of pro-inflammatory cytokine secretion in single-eye RPE, cells were treated with LPS, PIC, or TNF after a cultivation time of 14–18 d. Cells were stimulated for 1 or 3 d. As cell culture parameters, PDL vs. uncoated wells and 1%, 5%, and 10% serum were tested. Supernatants were applied in ELISA to detect secreted IL-8 (Figure 9).

##### IL-8 Secretion of Non-Polar Single-Eye Cultures

After 1 d of stimulation and using no coating with 1% serum, the control showed a secretion of 23.87 ± 71.22 pg/mL, and LPS increased IL-8 secretion up to 842.30 ± 890.61 pg/mL (*p* = 0.008). While using 10% serum without coating, the control showed secretion of 81.93 ± 101.95 pg/mL, while LPS and TNF increased IL-8 secretion to 2107.11 ± 1844.94 pg/mL (*p* = 0.040) and 2565.84 ± 933.21 pg/mL (*p* = 0.002), respectively. Using PDL as a coating together with 1% serum, all stressor agents increased IL-8 secretion compared to the control (34.08 ± 129.12 pg/mL), with LPS to 876.50 ± 669.03 pg/mL (*p* = 0.007), PIC to 326.87 ± 238.95 pg/mL (*p* = 0.028), and TNF to 721.85 ± 666.96 pg/mL (*p* = 0.030). Using 5% serum with PDL coating, the control showed a secretion of 0.00 ± 93.96 pg/mL, while LPS and TNF increased IL-8 secretion to 859.15 ± 554.12 pg/mL (*p* = 0.003) and 733.65 ± 673.34 pg/mL (*p* = 0.028), respectively.

After 3 d of stimulation using 1% serum without coating, the control showed a secretion of 89.59 ± 49.93 pg/mL, while secreted IL-8 was increased by LPS and PIC stimulation to 304.40 ± 814.87 pg/mL (*p* = 0.009) and 1332.66 ± 1923.51 pg/mL (*p* = 0.010), respectively. By using 5% serum without coating, the IL-8 level of control was 71.69 ± 126.35 pg/mL, which was elevated by LPS, PIC, and TNF to 2383.29 ± 3091.25 pg/mL (*p* = 0.020), 1686.62 ± 1939.59 pg/mL (*p* < 0.001), and 300.29 ± 557.98 pg/mL (*p* = 0.030). Using 10% serum without coating, the control showed a level of 70.50 ± 110.55 pg/mL, while LPS and TNF increased IL-8 secretion to 1681.40 ± 1934.55 pg/mL (*p* = 0.030) and 521.61 ± 1230.96 (*p* = 0.030). On the other hand, while using 1% serum with PDL, the control showed a basic secretion of 38.13 ± 97.03 pg/mL, while LPS, PIC, and TNF increased secreted IL-8 up to 1544.30 ± 1662.92 pg/mL (*p* = 0.002), 1730.00 ± 1509.76 pg/mL (*p* = 0.040), and 1460.34 ± 1144.95 pg/mL (*p* = 0.004), respectively. Using 5% serum together with PDL, the control level was 68.18 ± 76.01 pg/mL, and PIC and TNF elevated IL-8 secretion to 987.28 ± 869.01 pg/mL (*p* = 0.010) and 2350.84 ± 2073.10 pg/mL (*p* = 0.020), respectively. Using 10% serum with PDL coating, the control showed secretion of 110.26 ± 46.92 pg/mL, while these levels were increased with LPS and TNF to 1841.82 ± 2243.78 pg/mL (*p* = 0.030) and 297.35 ± 1397.77 pg/mL (*p* = 0.030).

##### IL-6 Secretion of Non-Polar Single-Eye Cultures

**Figure 10:**
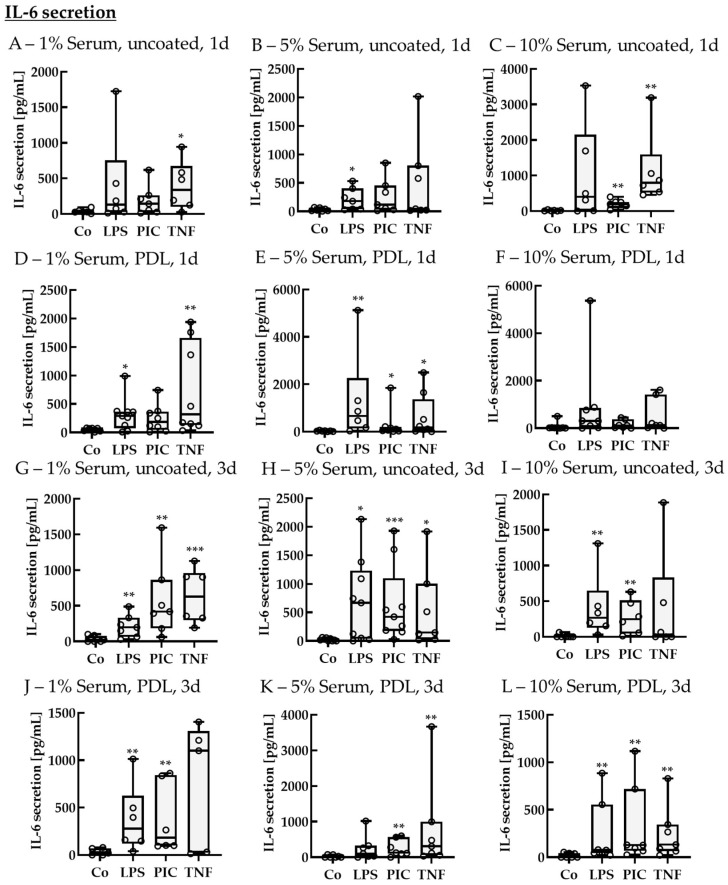
Interleukin 6 secretion of pro-inflammatorily activated porcine single-eye RPE cultures. Single-eye retinal pigment epithelium were stimulated on uncoated (**A**–**C**,**G**–**I**) or Poly-ᴅ-Lysine (PDL, (**D**–**F**,**J**–**L**)) coated 12-well plates with 1% (**A**,**D**,**G**,**J**), 5% (**B**,**E**,**H**,**K**), or 10% (**C**,**F**,**I**,**L**) serum-containing media. Cells were stimulated with 1 µg/mL lipopolysaccharide (LPS), 10 µg/mL polyinosinic/polycytidylic acid (Poly I:C, PIC), or 50 ng/mL tumor necrosis factor (TNF) for 1 (**A**–**F**) or 3 (**G**–**L**) d. Supernatants were collected and applied in ELISA to detect secreted interleukin 6 (IL-6). Data were not parametric. Significances were calculated with Kruskal–Wallis and Mann–Whitney tests against untreated control (Co). * *p* < 0.05, ** *p* < 0.01, *** *p* < 0.01, *n* = 6–9. Circles = individual data points.

Supernatants were also analyzed for IL-6 secretion (Figure 10). In general, the cells secreted less IL-6 than IL-8. After 1 d of stimulation, using 1% serum without coating, the control level was 27.37 ± 43.26 pg/mL, while TNF increased IL-6 secretion to 338.06 ± 416.49 pg/mL (*p* = 0.040). Using 5% serum without coating, the control was at 22.87 ± 40.91 pg/mL, while LPS increased IL-6 levels to 180.38 ± 279.92 pg/mL (*p* = 0.040). For 10% serum without coating, the control showed secretion of 12.78 ± 14.49 pg/mL, and PIC and TNF increased IL-6 secretion to 193.49 ± 139.16 pg/mL (*p* = 0.002) and 794.73 ± 424.15 pg/mL (*p* = 0.002), respectively. Using PDL coating with 1% serum, the control level was 69.85 ± 46.08 pg/mL, while IL-6 secretion was elevated by LPS and TNF up to 290.25 ± 236.06 pg/mL (*p* = 0.040) and 317.63 ± 1314.53 pg/mL (*p* = 0.004), respectively. Using 5% serum with PDL coating, the control showed values of 23.38 ± 20.68 pg/mL, while LPS, PIC, and TNF increased IL-6 secretion to 662.54 ± 950.44 pg/mL (*p* = 0.005), 85.26 ± 131.20 pg/mL (*p* = 0.030), and 169.63 ± 736.98 pg/mL (*p* = 0.030), respectively. Using 10% serum with PDL did not show any significant effects. After 3 d of stimulation, using 1% serum without coating, the control level was 23.52 ± 63.36 pg/mL, and LPS, PIC, and TNF increased IL-6 levels to 197.24 ± 176.65 pg/mL (*p* = 0.006), 419.03 ± 398.50 pg/mL (*p* = 0.001), and 629.97 ± 571.07 pg/mL (*p* < 0.001), respectively. Using 5% serum without coating, the control showed secretion of 18.72 ± 43.32 pg/mL IL-6, while LPS, PIC, and TNF increased IL-6 secretion to 669.36 ± 1041.49 pg/mL (*p* = 0.010), 425.00 ± 410.76 pg/mL (*p* < 0.001), and 146.22 ± 682.61 pg/mL (*p* = 0.010), respectively. Using 10% serum without coating, the control level was 0.00 ± 8.44 pg/mL, while LPS and PIC increased IL-6 levels to 266.73 ± 241.58 pg/mL (*p* = 0.001) and 247.43 ± 331.19 pg/mL (*p* = 0.004), respectively. Using 1% serum with PDL coating, the control level was 26.44 ± 54.95 pg/mL, while LPS and PIC elevated IL-6 to 278.16 ± 322.20 pg/mL (*p* = 0.009) and 183.38 ± 593.09 pg/mL (*p* = 0.002), respectively. Using 5% serum with PDL, the control level was 0.00 ± 50.44 pg/mL, while PIC and TNF increased IL-6 secretion to 128.37 ± 314.78 pg/mL (*p* = 0.010) and 307.23 ± 649.14 pg/mL (*p* = 0.006), respectively. Using 10% serum with PDL, the control level was 0.00 ± 41.47 pg/mL, while LPS, PIC, and TNF increased IL-6 levels to 76.07 ± 271.11 pg/mL (*p* = 0.009), 128.42 ± 342.85 pg/mL (*p* = 0.004), and 133.38 ± 228.18 pg/mL (*p* = 0.004), respectively.

##### TNF Secretion of Non-Polar Single-Eye Cultures

**Figure 11:**
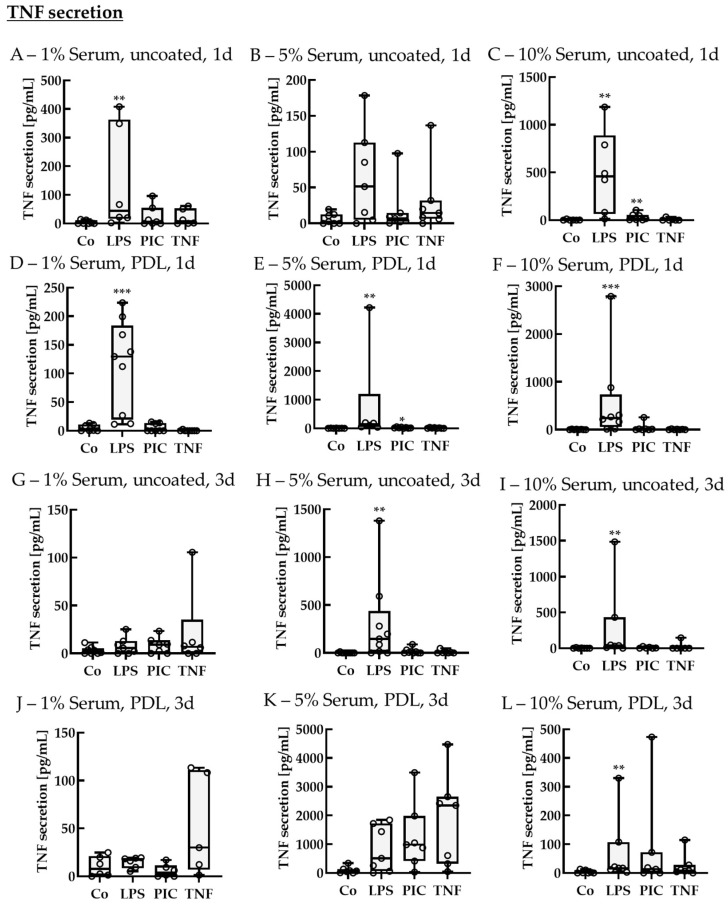
Tumor necrosis factor secretion of pro-inflammatorily activated porcine single-eye RPE cultures. Single-eye retinal pigment epithelium were stimulated on uncoated (**A**–**C**,**G**–**I**) or Poly-ᴅ-Lysine (PDL, (**D**–**F**,**J**–**L**)) coated 12-well plates with 1% (**A**,**D**,**G**,**J**), 5% (**B**,**E**,**H**,**K**), or 10% (**C**,**F**,**I**,**L**) serum-containing media. Cells were stimulated with 1 µg/mL lipopolysaccharide (LPS), 10 µg/mL polyinosinic/polycytidylic acid (Poly I:C, PIC), or 50 ng/mL tumor necrosis factor (TNF) for 1 (**A**–**F**) or 3 (**G**–**L**) d. Supernatants were collected and applied in ELISA to detect secreted tumor necrosis factor alpha (TNF). Data were not parametric. Significances were calculated with Kruskal–Wallis and Mann–Whitney tests against untreated control (Co). * *p* < 0.05, ** *p* < 0.01, *** *p* < 0.01, *n* = 6–9. Circles = individual data points.

Finally, supernatants were analyzed for TNF secretion (Figure 11). In general, secreted TNF content was not as high as IL-6 or IL-8. After 1 d of stimulation, using 1% serum without coating, TNF secretion of control was 0.68 ± 7.24 pg/mL and was upregulated by LPS to 44.56 ± 257.48 pg/mL (*p* = 0.005). Using 5% serum without coating exhibited no significant effects, but when using 10% serum without coating, the control level was 0.64 ± 1.96 pg/mL, while LPS and PIC increased TNF secretion to 457.59 ± 547.29 pg/mL (*p* = 0.002) and 15.68 ± 45.42 pg/mL (*p* = 0.008), respectively. Using 1% serum with PDL coating, the control level was 0.82 ± 9.65 pg/mL, while LPS increased TNF secretion to 129.73 ± 141.24 pg/mL (*p* < 0.001). Using 5% serum with PDL, the control level was 2.20 ± 7.47 pg/mL, while LPS and PIC increased TNF secretion to 143.23 ± 128.31 pg/mL (*p* = 0.001) and 16.95 ± 21.09 pg/mL (*p* = 0.020), respectively. Using 10% serum and PDL, the control showed a secretion of 0.18 ± 9.88 pg/mL, while only LPS increased TNF secretion to 243.47 ± 319.88 pg/mL (*p* < 0.001). After 3 d of stimulation, 1% serum without coating did not show any significant effects, but for 5% serum without coating, the control was at 0.00 ± 0.66 pg/mL, while LPS increased TNF secretion to 146.44 ± 263.86 pg/mL (*p* = 0.002). Also, with 10% serum without coating, control was at a level of 0.00 ± 1.96 pg/mL, while LPS elevated TNF levels to 37.45 ± 218.15 pg/mL (*p* = 0.010). Using 1% or 5% serum with PDL coating did not show any significant changes in TNF levels, while for 10% serum with PDL, the control had a level of 1.10 ± 6.06 pg/mL, while LPS increased secreted TNF to 16.88 ± 50.25 pg/mL (*p* = 0.003). Taken together, LPS is a potent activator for IL-8, IL-6, and TNF in single-eye RPE. Recombinant TNF used for stimulation did not increase TNF secretion. PDL is optional and not needed as a coating; furthermore, a reduction to 5% serum is practicable. Also, 3 d of stimulation leads to higher and more efficient results.

#### 2.2.3. Cytokine Secretion—Polar

**Figure 12:**
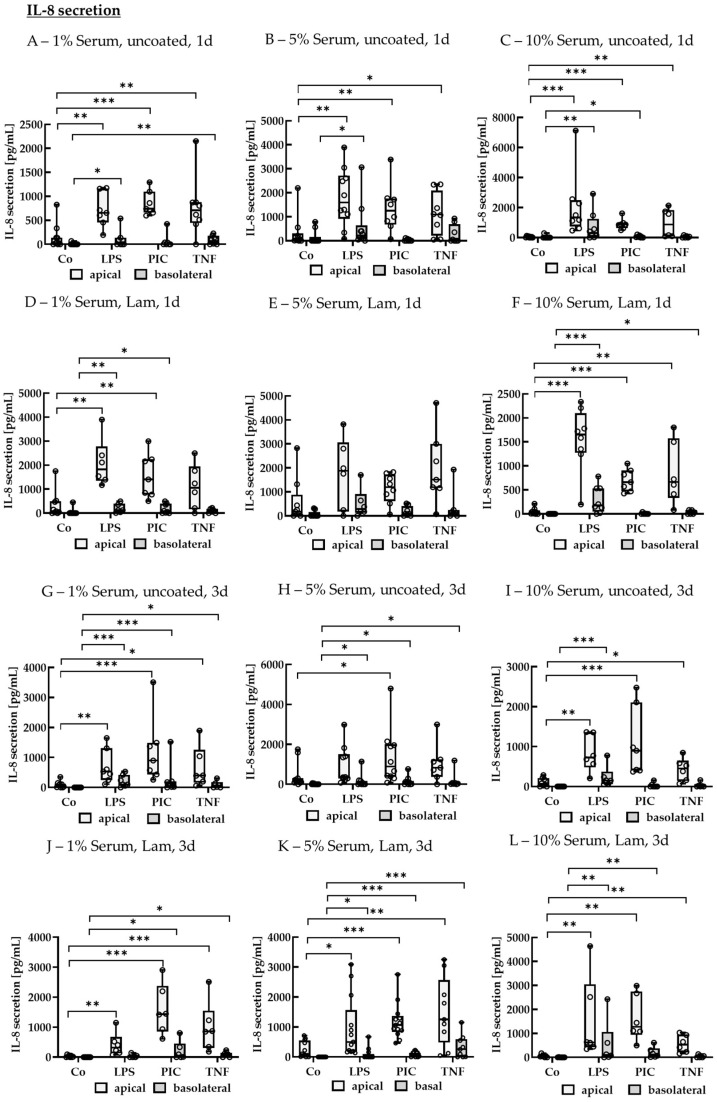
Interleukin 8 secretion of pro-inflammatorily activated polar single-eye RPE cultures. Single-eye retinal pigment epithelium were stimulated on uncoated (**A**–**C**,**G**–**I**) or laminin (Lam, (**D**–**F**,**J**–**L**)) coated transwell inserts with 1% (**A**,**D**,**G**,**J**), 5% (**B**,**E**,**H**,**K**), or 10% (**C**,**F**,**I**,**L**) serum-containing media. Cells were stimulated with 1 µg/mL lipopolysaccharide (LPS), 10 µg/mL polyinosinic/polycytidylic acid (Poly I:C, PIC), or 50 ng/mL tumor necrosis factor (TNF) for 1 (**A**–**F**) or 3 (**G**–**L**) d. Apical and basolateral supernatants were collected and applied in ELISA to detect secreted interleukin 8 (IL-8). Data were not parametric. Significances were calculated with Kruskal–Wallis and Mann–Whitney tests against untreated control (Co). * *p* < 0.05, ** *p* < 0.01, *** *p* < 0.01, *n* = 6–13. Circles = individual data points.

To test the induction of pro-inflammatory cytokine secretion in polar single-eye RPE, cells were treated with LPS, PIC, or TNF after a cultivation time of 14–18 d. Cells were stimulated for 1 or 3 d. As cell culture parameters, Lam-coated vs. Lam-uncoated wells and 1%, 5%, or 10% serum were tested. Apical and basolateral supernatants were applied in ELISA to detect secreted IL-8 (Figure 12).

##### IL-8 Secretion of Polar Single-Eye Cultures

After 1 d of stimulation, using no coating with 1% serum, LPS, PIC, and TNF increased apical IL-8 secretion to 655.67 ± 405.93 pg/mL (*p* = 0.002), 741.16 ± 296.39 pg/mL (*p* < 0.001), and 710.17 ± 384.87 pg/mL (*p* = 0.009) compared to control with 27.19 ± 124.43 pg/mL, while basolaterally, TNF increased IL-8 secretion to 62.54 ± 139.06 pg/mL (*p* = 0.003) compared to control with 0.00 ± 0.00 pg/mL. Using 5% serum without coating, LPS, PIC, and TNF increased apical IL-8 secretion to 1597.03 ± 1425.97 pg/mL (*p* = 0.002), 1253.60 ± 963.49 pg/mL (*p* = 0.008), and 1090.82 ± 1087.76 pg/mL (*p* = 0.030) compared to control with 88.43 ± 180.76 pg/mL, while basolaterally, only LPS significantly increased IL-8 secretion to 214.22 ± 312.42 pg/mL (*p* = 0.020) compared to control with 0.00 ± 18.49 pg/mL. Using 10% serum without coating led to an increased apical secretion of IL-8 by LPS, PIC, and TNF to 1335.45 ± 1351.91 pg/mL (*p* < 0.001), 873.11 ± 193.23 pg/mL (*p* < 0.001), and 868.96 ± 1590.67 pg/mL (*p* = 0.003), respectively, compared to control with 53.02 ± 63.62 pg/mL, while basolaterally, LPS and PIC exhibited an increased IL-8 secretion of 303.61 ± 672.70 pg/mL (*p* = 0.002) and 40.42 ± 79.03 pg/mL (*p* = 0.030) compared to control with 0.00 ± 0.00 pg/mL. Using Lam coating with 1% serum, LPS and PIC increased apical IL-8 secretion to 1828.32 ± 901.55 pg/mL (*p* = 0.003) and 1404.18 ± 1389.55 pg/mL (*p* = 0.003), respectively, compared to control with 78.79 ± 436.45 pg/mL, while basolateral secretion was increased by LPS and PIC to 122.82 ± 233.66 pg/mL (*p* = 0.006) and 71.10 ± 349.68 pg/mL (*p* = 0.020) compared to control with 0.00 ± 0.00 pg/mL. Lam coating with 5% serum showed no significant changes with any stressor, while 10% serum with Lam showed increased apical secretion by LPS, PIC, and TNF with 1651.84 ± 560.73 pg/mL (*p* < 0.001), 661.58 ± 341.54 pg/mL (*p* < 0.001), and 665.33 ± 842.37 pg/mL (*p* = 0.002), respectively, compared to control with 25.67 ± 39.28 pg/mL, while basolateral secretion was elevated by LPS and TNF to 177.27 ± 430.30 pg/mL (*p* < 0.001) and 16.17 ± 67.95 pg/mL (*p* = 0.040) compared to control with 0.00 ± 0.00 pg/mL.

After 3 d of stimulation, using 1% serum without coating, LPS, PIC, and TNF increased apical IL-8 secretion to 532.24 ± 606.40 pg/mL (*p* = 0.002), 899.80 ± 926.07 pg/mL (*p* < 0.001), and 396.36 ± 637.53 pg/mL (*p* = 0.010) compared to control with 83.24 ± 101.58 pg/mL, while basolateral secretion was lower but still significantly elevated, with LPS, PIC, and TNF increasing it to 98.25 ± 252.84 pg/mL (*p* < 0.001), 122.48 ± 172.44 pg/mL (*p* < 0.001), and 49.44 ± 116.22 pg/mL (*p* = 0.010), respectively, compared to control with 0.00 ± 0.00 pg/mL. Using 5% serum without coating, only PIC increased apical IL-8 secretion significantly to 891.29 ± 1538.44 pg/mL (*p* = 0.010) compared to control with 124.48 ± 166.27 pg/mL, while basolaterally, all stressors, LPS, PIC, and TNF, significantly increased secretion to 28.13 ± 142.92 pg/mL (*p* = 0.040), 83.65 ± 120.09 pg/mL (*p* = 0.010), and 32.64 ± 51.36 pg/mL (*p* = 0.010) compared to control with 0.00 ± 3.27 pg/mL. Using 10% serum without coating, LPS, PIC, and TNF increased apical IL-8 secretion to 727.18 ± 613.28 pg/mL (*p* = 0.003), 899.65 ± 1132.06 pg/mL (*p* < 0.001), and 451.89 ± 351.78 pg/mL (*p* = 0.030) compared to control with 63.87 ± 173.89 pg/mL, while basolaterally, only LPS increased secretion to 152.25 ± 128.13 pg/mL (*p* < 0.001) compared to control with 0.00 ± 0.00 pg/mL. Using Lam coating and 1% serum, LPS, PIC, and TNF increased apical IL-8 secretion to 314.70 ± 325.89 pg/mL (*p* = 0.001), 1435.77 ± 953.56 pg/mL (*p* < 0.001), and 861.38 ± 670.46 pg/mL (*p* < 0.001) compared to control with 15.31 ± 54.08 pg/mL, while basolateral secretion was increased by PIC and TNF to 53.17 ± 271.76 pg/mL (*p* = 0.010) and 92.72 ± 84.32 pg/mL (*p* = 0.010) compared to control with 0.00 ± 0.00 pg/mL. Using Lam coating with 5% serum, LPS, PIC, and TNF increased apical IL-8 secretion to 501.83 ± 871.52 pg/mL (*p* = 0.010), 1067.38 ± 406.63 pg/mL (*p* < 0.001), and 1254.35 ± 1243.02 pg/mL (*p* = 0.002), respectively, compared to control with 87.00 ± 463.05 pg/mL, while basolaterally, PIC and TNF elevated IL-8 secretion to 60.59 ± 112.76 pg/mL (*p* < 0.001) and 253.53 ± 544.52 pg/mL (*p* < 0.001) compared to control with 0.00 ± 0.00 pg/mL. Using Lam coating with 10% serum, LPS, PIC, and TNF increased apical IL-8 secretion to 625.05 ± 1558.31 pg/mL (*p* = 0.001), 1278.52 ± 1274.95 pg/mL (*p* = 0.001), and 532.05 ± 584.12 pg/mL (*p* = 0.002), respectively, compared to control with 47.23 ± 36.33 pg/mL, while basolaterally LPS and PIC elevated IL-8 to 103.03 ± 452.00 pg/mL (*p* = 0.009) and 116.34 ± 224.38 pg/mL (*p* = 0.002) compared to control with 0.00 ± 0.00 pg/mL.

##### IL-6 Secretion of Polar Single-Eye Cultures

**Figure 13:**
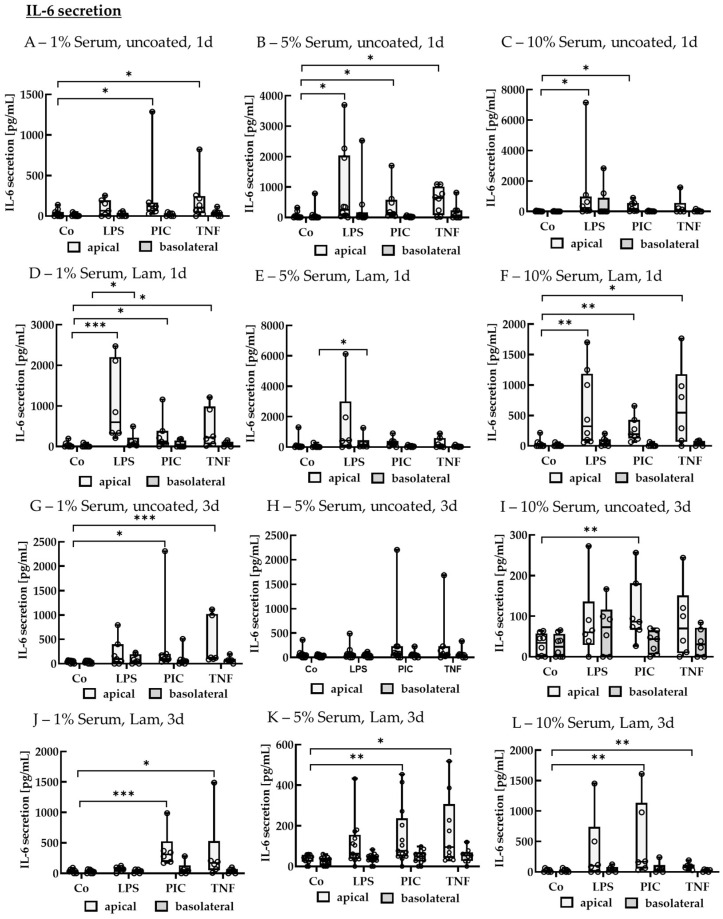
Interleukin 6 secretion of pro-inflammatorily activated polar single-eye RPE cultures. Single-eye retinal pigment epithelium were stimulated on uncoated (**A**–**C**,**G**–**I**) or laminin (Lam, (**D**–**F**,**J**–**L**)) coated transwell inserts with 1% (**A**,**D**,**G**,**J**), 5% (**B**,**E**,**H**,**K**), or 10% (**C**,**F**,**I**,**L**) serum-containing media. Cells were stimulated with 1 µg/mL lipopolysaccharide (LPS), 10 µg/mL polyinosinic/polycytidylic acid (Poly I:C, PIC), or 50 ng/mL tumor necrosis factor (TNF) for 1 (**A**–**F**) or 3 (**G**–**L**) d. Apical and basolateral supernatants were collected and applied in ELISA to detect secreted interleukin 6 (IL-6). Data were not parametric. Significances were calculated with Kruskal–Wallis and Mann–Whitney tests against untreated control (Co). * *p* < 0.05, ** *p* < 0.01, *** *p* < 0.01, *n* = 6–13. Circles = individual data points.

Supernatants were also analyzed for IL-6 secretion (Figure 13). After 1 d of stimulation, using 1% serum without coating, PIC and TNF increased apical IL-6 secretion to 119.67 ± 86.76 pg/mL (*p* = 0.010) and 102.31 ± 169.96 pg/mL (*p* = 0.030) compared to the control with 24.43 ± 41.62 pg/mL, while no significant elevated basolateral secretion was detected. Using 5% serum without coating, LPS, PIC, and TNF increased apical IL-6 secretion to 251.35 ± 1498.19 pg/mL (*p* = 0.020), 147.46 ± 442.08 pg/mL (*p* = 0.006), and 642.20 ± 672.73 pg/mL (*p* = 0.006) compared to the control with 17.77 ± 39.38 pg/mL, while on the basolateral side no significant effects were detected. Using 10% without serum, LPS and PIC increased IL-6 levels to 227.53 ± 685.63 pg/mL (*p* = 0.005) and 169.61 ± 485.93 pg/mL (*p* = 0.005) compared to the control with 0.00 ± 27.98 pg/mL with no significant basolateral effects. Using Lam coating with 1% serum, LPS, PIC, and TNF increased apical IL-6 secretion to 595.09 ± 1458.36 pg/mL (*p* < 0.001), 123.57 ± 236.91 pg/mL (*p* = 0.020), and 222.70 ± 647.37 pg/mL (*p* = 0.020) compared to control with 21.76 ± 40.29 pg/mL, while basolaterally, LPS increased secretion to 67.69 ± 78.24 pg/mL (*p* = 0.020) compared to control with 0.00 ± 31.92 pg/mL. Using Lam coating with 5% serum, no significant apical effects were determined, but LPS increased basolateral IL-6 secretion to 123.17 ± 144.70 pg/mL (*p* = 0.030) compared to the control with 21.87 ± 44.68 pg/mL. Using Lam with 10% serum, LPS, PIC, and TNF increased apical IL-6 secretion to 322.68 ± 966.11 pg/mL (*p* = 0.001), 190.40 ± 213.48 pg/mL (*p* = 0.002), and 547.81 ± 798.62 pg/mL (*p* = 0.010), respectively, compared to control with 0.00 ± 39.86 pg/mL, while no significant basolateral effects were detected.

After 3 d of stimulation, using 1% serum without coating, PIC and TNF increased apical IL-6 secretion to 95.94 ± 117.34 pg/mL (*p* = 0.020) and 123.29 ± 674.86 pg/mL (*p* < 0.001) compared to control with 55.90 ± 47.56 pg/mL with no significant basolateral effects. Using 5% serum without coating, no significant effects were detected. Using 10% serum without coating, PIC increased apical IL-6 secretion to 86.00 ± 67.47 pg/mL (*p* = 0.004) compared to the control with 34.02 ± 48.32 pg/mL, while no significant basolateral effects were detected. Using Lam coating with 1% serum, PIC and TNF increased apical IL-6 secretion to 309.61 ± 150.89 pg/mL (*p* < 0.001) and 169.69 ± 114.67 pg/mL (*p* = 0.030) compared to control with 39.74 ± 29.17 pg/mL, while no significant basolateral effects were achieved. Using Lam coating with 5% serum, PIC and TNF increased IL-6 secretion to 76.57 ± 153.65 pg/mL (*p* = 0.002) and 94.55 ± 176.64 pg/mL (*p* = 0.010) compared to the control with 41.46 ± 24.77 pg/mL with no basolateral effects detected. Using Lam coating with 10% serum, PIC and TNF increased apical IL-6 secretion to 166.88 ± 689.31 pg/mL (*p* = 0.005) and 87.30 ± 23.86 pg/mL (*p* = 0.008) compared to control with 26.28 ± 35.67 pg/mL with no significant basolateral effects detected.

##### TNF Secretion of Polar Single-Eye Cultures

**Figure 14:**
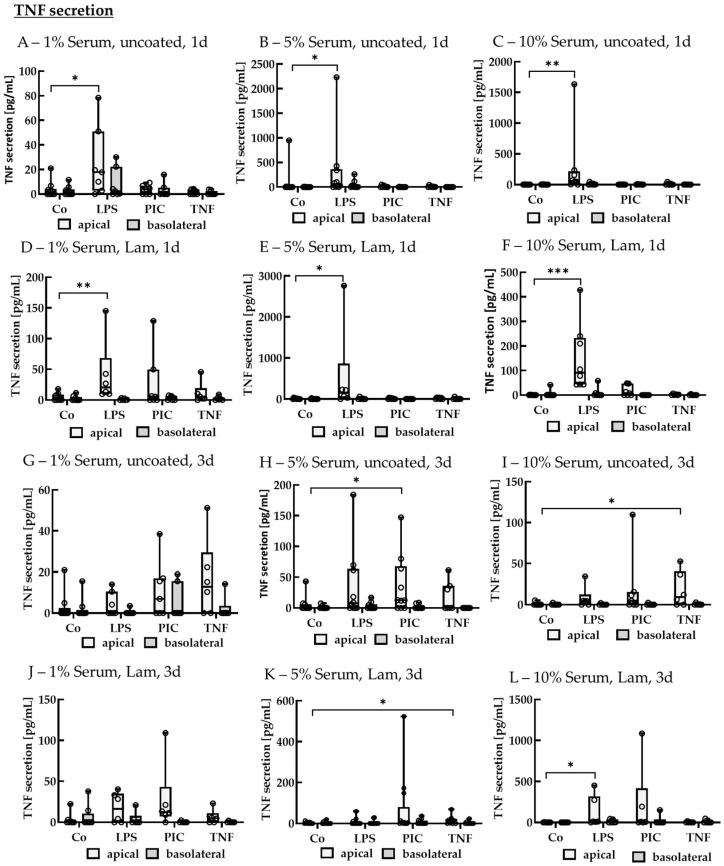
Tumor necrosis factor secretion of pro-inflammatorily activated polar single-eye RPE cultures. Single-eye retinal pigment epithelium were stimulated on uncoated (**A**–**C**,**G**–**I**) or laminin (Lam, **D**–**F**,**J**–**L**) coated transwell inserts with 1% (**A**,**D**,**G**,**J**), 5% (**B**,**E**,**H**,**K**), or 10% (**C**,**F**,**I**,**L**) serum-containing media. Cells were stimulated with 1 µg/mL lipopolysaccharide (LPS), 10 µg/mL polyinosinic/polycytidylic acid (Poly I:C, PIC), or 50 ng/mL tumor necrosis factor (TNF) for 1 (**A**–**F**) or 3 (**G**–**L**) d. Apical and basolateral supernatants were collected and applied in ELISA to detect secreted tumor necrosis factor alpha (TNF). Data were not parametric. Significances were calculated with Kruskal–Wallis and Mann–Whitney tests against untreated control (Co). * *p* < 0.05, ** *p* < 0.01, *** *p* < 0.01, *n* = 6–13. Circles = individual data points.

Supernatants were also analyzed for TNF secretion (Figure 14). After 1 d of stimulation, using 1% serum without coating, LPS increased apical TNF secretion to 17.79 ± 28.88 pg/mL (*p* = 0.030) compared to the control with 1.15 ± 3.21 pg/mL. Using 5% serum without coating, LPS increased apical TNF secretion to 57.13 ± 270.69 pg/mL (*p* = 0.030) compared to control with 0.00 ± 2.89 pg/mL, while for 10% serum, LPS increased apical TNF secretion to 61.78 ± 141.19 pg/mL (*p* = 0.002) compared to control with 0.00 ± 2.07 pg/mL. Using Lam coating with 1% serum, LPS increased apical TNF secretion to 20.25 ± 27.85 pg/mL (*p* = 0.007) compared to control with 1.70 ± 7.58 pg/mL, while for 5% serum, TNF was increased by LPS to 147.15 ± 187.99 pg/mL (*p* = 0.020) compared to control with 1.31 ± 13.10 pg/mL, and for 10% serum, TNF was increased by LPS to 0.00 ± 0.36 pg/mL (*p* < 0.001). No significant upregulation of TNF was detected on the basolateral side.

After 3 d of stimulation, using 5% serum without coating, PIC increased apical TNF secretion to 12.57 ± 53.17 pg/mL (*p* = 0.030) compared to the control with 0.00 ± 4.22 pg/mL. Using 10% serum without coating, TNF increased apical TNF secretion to 9.21 ± 28.98 pg/mL (*p* = 0.040) compared to the control with 0.00 ± 0.00 pg/mL. Using 5% serum with Lam coating, TNF increased TNF secretion to 14.20 ± 17.64 pg/mL (*p* = 0.040) compared to control with 0.00 ± 2.77 pg/mL, while using 10% serum with Lam coating, LPS increased apical TNF secretion to 10.63 ± 201.60 pg/mL (*p* = 0.010) compared to control with 0.00 ± 0.04 pg/mL. Other than those, no significant TNF changes were determined.

#### 2.2.4. Gene Expression

To assess inflammation-related gene expression, primary porcine RPE of single-eye cultures were treated with the best parameters from Section 2.2.2. For this, 1 µg/mL LPS and 10 µg/mL PIC were used for 3 d stimulation on 12-well plates not coated or coated with PDL using 10% or 5% serum. RNA was isolated, and RT-qPCR was performed. *GUSB* was used as an endogenous control. Targets for inflammation were *IL1B*, *IL6*, *CXCL8,* and *NOS2*. Data were evaluated with Thermo Fisher Connect and set in relation to the untreated control (Rq = 1.00) (Table 2).

LPS and PIC did not influence gene expression as effectively as protein secretion. Especially after using PDL (with 5% serum), there were no significant changes in inflammation-related genes. Using 10% serum without coating, there was one significant finding. Here, LPS stimulation led to an increased gene expression of *IL1B* (Rq = 8.04, *p* = 0.035). Gene expression of *IL6* and *CXCL8,* on the other hand, was changed by using 5% serum without coating, as PIC unexpectedly decreased gene expression significantly (Rq = 0.44, *p* = 0.048 and Rq = 0.40, *p* = 0.036). Considering LPS with 5% serum and no coating, there were no significant findings concerning gene expression. If inflammation studies with gene expression should be conducted, one might choose 10% serum and LPS to activate *IL1B* expression, or tests for shorter stimulation times should be conducted.

Experiments were repeated with polar single-eye cultures on transwell inserts using Lam as an optional coating (Table 3). Using 5% serum, PIC reduced gene expression of *IL6* (Rq = 0.33, *p* = 0.042) and *NOS2* (Rq = 0.21, *p* = 0.040) significantly, while with 10% serum, PIC did not lead to any significant gene alteration. For 5% serum with Lam coating, again, PIC unexpectedly reduced *NOS2* (Rq = 0.16, *p* = 0.022) gene expression, but LPS significantly increased *CXCL8* (Rq = 2.84, *p* = 0.006) gene expression. Regarding transwell approaches for gene expression studies, 5% serum and Lam coating with LPS could be used to investigate *CXCL8* gene expression.

#### 2.2.5. Barrier

**Figure 15:**
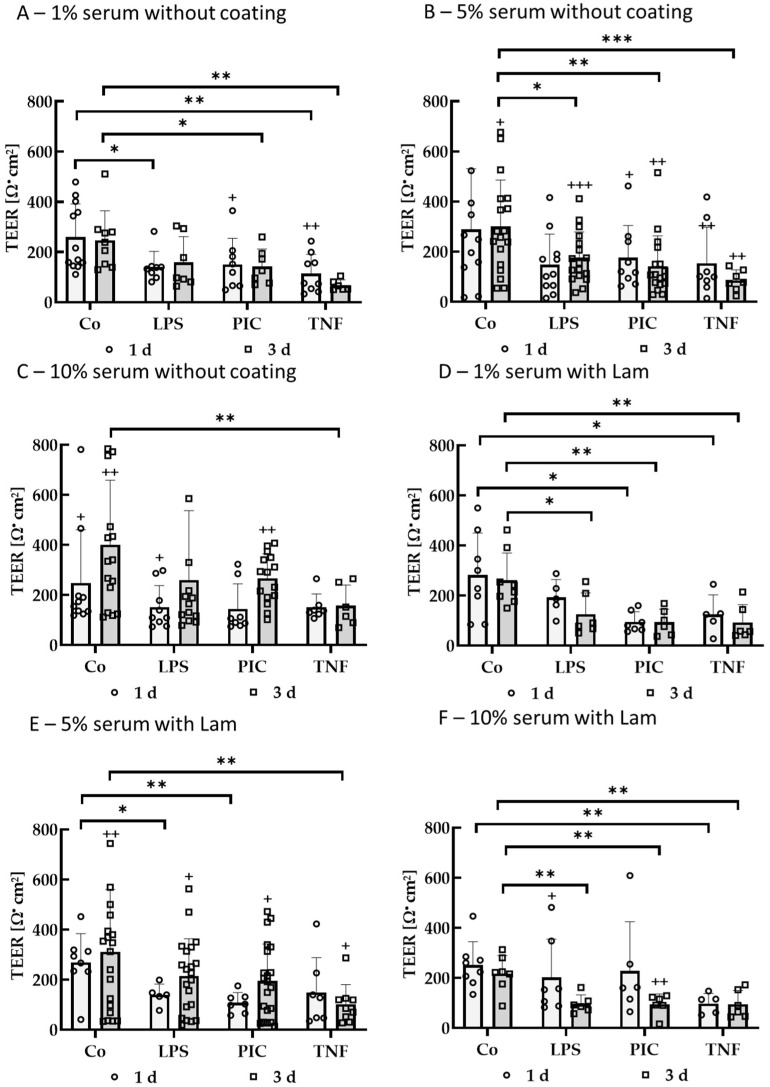
Cell barrier of pro-inflammatorily activated polar single-eye RPE cultures. Single-eye retinal pigment epithelium cultures were stimulated on uncoated (**A**–**C**) or laminin (Lam, **D**–**F**) coated 12-well plates with transwell inserts with 1% (**A**,**D**), 5% (**B**,**E**), or 10% (**C**,**F**) serum-containing media. Cells were stimulated with 1 µg/mL lipopolysaccharide (LPS), 10 µg/mL polyinosinic/polycytidylic acid (Poly I:C, PIC), or 50 ng/mL tumor necrosis factor (TNF) for 1 or 3 d (separately, not the same well). Cell barrier was determined with transepithelial electrical resistance (TEER) measurement. Data were parametric. Significances were calculated with analysis of variance (ANOVA) and Student’s *t*-test. * *p* < 0.05, ** *p* < 0.01, *** *p* < 0.001 against untreated control Co; + *p* < 0.05, ++ *p* < 0.01, +++ *p* < 0.001 against pre-stimulation TEER. *n* = 5–21.

To test the influence of pro-inflammatory insults on the single-eye RPE cell barrier, cells on transwell inserts were treated with LPS, PIC, or TNF after a cultivation time of 14–18 d. Cells were stimulated for 1 or 3 d. As cell culture parameters, Lam vs. uncoated transwells and 1%, 5%, and 10% serum were tested. Cell barrier was determined by TEER measurement (Figure 15). Significances were calculated against untreated controls or pre-stimulation TEER. Summarized TEER data of pre-stimulation (day 0) are listed (Table 4).

After stimulation, using 1% serum without coating, PIC and TNF were decreasing TEER compared to pre-stimulation (*p* = 0.048 and *p* = 0.002), and LPS and PIC reduced the barrier to 141.70 ± 60.86 Ω·cm^2^ (*p* = 0.016) and 114.73 ± 75.81 Ω·cm^2^ (*p* = 0.005) compared to control (259.18 ± 132.34 Ω·cm^2^) after 1 day of stimulation. After 3 d of stimulation, PIC and TNF reduced the barrier to 142.16 ± 69.89 Ω·cm^2^ (*p* = 0.044) and 67.88 ± 21.57 Ω·cm^2^ (*p* = 0.002) compared to the control (246.94 ± 117.05 Ω·cm^2^). Using 5% serum without coating, PIC lowered the cell barrier compared to pre-stimulation (*p* = 0.033) after 1 d of stimulation, whereas after 3 d of stimulation, LPS, PIC, and TNF decreased the barrier compared to pre-stimulation (*p* < 0.001, *p* = 0.003, and *p* = 0.009, respectively), but also the TEER of the control well was reduced (*p* = 0.018). Also, LPS, PIC, and TNF significantly reduced the barrier to 175.32 ± 102.49 Ω·cm^2^ (*p* = 0.020), 142.31 ± 120.35 Ω·cm^2^ (*p* = 0.006), and 85.21 ± 42.12 Ω·cm^2^ (*p* < 0.001), respectively, compared to the control (299.76 ± 185.71 Ω·cm^2^). Using 10% serum without coating, after 1 d of stimulation, the control barrier (*p* = 0.024) and the LPS (*p* = 0.032) treated cell barrier were significantly lower than pre-stimulation. After 3 d of stimulation, the control barrier (*p* = 0.001) and PIC (*p* = 0.004) treated cell barrier were lower than pre-stimulation, and TNF reduced the barrier significantly to 157.28 ± 82.96 Ω·cm^2^ (*p* = 0.003) compared to control (400.33 ± 258.54 Ω·cm^2^). Using 1% serum with Lam coating, after 1 d of stimulation, PIC and TNF reduced the barrier to 94.81 ± 40.63 Ω·cm^2^ (*p* = 0.016) and 124.88 ± 78.38 Ω·cm^2^ (*p* = 0.044) compared to the control (282.01 ± 167.60 Ω·cm^2^). After 3 d of stimulation, LPS, PIC, and TNF lowered the barrier to 125.52 ± 85.24 Ω·cm^2^ (*p* = 0.023), 94.07 ± 52.04 Ω·cm^2^ (*p* = 0.003), and 92.85 ± 70.75 Ω·cm^2^ (*p* = 0.005), respectively, compared to the control (260.96 ± 109.34 Ω·cm^2^). Using 5% serum with Lam coating, after 1 d of stimulation, LPS and PIC reduced cell barrier to 139.52 ± 43.09 Ω·cm^2^ (*p* = 0.017) and 108.40 ± 40.75 Ω·cm^2^ (*p* = 0.005) compared to control (269.29 ± 115.17 Ω·cm^2^). After 3 d of stimulation, TEER of control (*p* = 0.001), LPS (*p* = 0.042), PIC (*p* = 0.019), and TNF (*p* = 0.022) was reduced compared to pre-stimulation, and also TNF reduced the barrier significantly to 102.01 ± 78.93 Ω·cm^2^ (*p* = 0.002) compared to control (311.63 ± 248.82 Ω·cm^2^). Using 10% serum with Lam coating, after 1 d of stimulation, the LPS cell barrier was reduced compared to pre-stimulation (*p* = 0.032), and TNF reduced it to 98.44 ± 40.33 Ω·cm^2^ (*p* = 0.002) compared to the control (251.28 ± 93.50 Ω·cm^2^). After 3 d of stimulation, the cell barrier of PIC-treated wells was reduced compared to pre-stimulation (*p* = 0.010), and LPS, PIC, and TNF reduced the barrier to 96.35 ± 36.58 Ω·cm^2^ (*p* = 0.004), 94.57 ± 41.51 Ω·cm^2^ (*p* = 0.004), and 95.22 ± 55.32 Ω·cm^2^ (*p* = 0.006), respectively, compared to the control (217.24 ± 72.54 Ω·cm^2^). Taken together, LPS stimulation for 3 d with 5% serum without coating seems to be a good choice for testing influence on the barrier under pro-inflammatory conditions, as the barrier was reduced significantly compared to the untreated control and to pre-stimulation.

## 3. Discussion

In this study, we have established optimized protocols for the investigation of oxidative stress and pro-inflammatory activation in single-eye primary porcine RPE cultures. We have recently published a best-practice protocol for cultivation of single-eye primary porcine RPE cells, allowing a reproducible and relatively easy-to-handle RPE cell model with genetically homogeneous cells within and genetically heterogeneous cells between cultures, containing highly differentiated RPE cells. These cultures are prepared from porcine eyes, obtained as slaughterhouse waste material, thereby combining cells of a species with a high homology to the human situation (pig) with the 3R principle of reducing the burden of animal experimentation [3,4]. As oxidative stress and inflammation are important in retinal degenerative diseases, e.g., in age-related macular degeneration [10,17,18,19], we aimed for establishing reproducible and easy-to-handle protocols for investigating oxidative stress and inflammation in our RPE cell culture model.

An obstacle in inducing oxidative stress in primary RPE is the fact that these cells are inherently very resistant to oxidative stress, resistance mediated via Nrf2 [20,21]. Therefore, for this approach, we tried out different cultivation times in order to find more susceptible cultures. In addition, we aimed for a reduction in viability of about 50% in order to have a suitable loss of viability to be able to detect protection but to avoid a full-scale death of the culture, which would make any attempt to save the cells very difficult.

We have investigated different pathways of oxidative stress induction: direct oxidative stress, using H_2_O_2_ and NaIO_3_ [22]; indirect oxidative stress, using erastin [23]; and oxidative stress via hypoxia, using CoCl_2_ [24]. H_2_O_2_ is a reactive oxygen species that, in lower concentrations, participates in physiological signal transduction. It is freely diffusible (through aquaporins) across the cell membrane and is detoxified by catalase, superoxide dismutase, and glutathione. However, in high concentrations, H_2_O_2_ can induce irreversible posttranslational modifications at cellular molecules and cell death, which, depending on the concentration, can be apoptotic, necroptotic, or necrotic [22,25,26]. It is widely used in studies concerning oxidative stress-induced cell death in the RPE [e.g., [27,28,29] and many more]. Of note, these studies are mainly conducted in ARPE-19 cells and less in primary RPE cells, most likely because of the high resistance of primary RPE cells to H_2_O_2_ stimulation, which is mediated by Nrf2 [20]. Accordingly, the amount needed to induce reproducible cell death in our primary porcine single-eye RPE cells is rather high (500 µM). Of note, H_2_O_2_ is not very stable, losing its potency over time, making reproducibility hard to achieve.

NaIO_3_ is a powerful oxidant that primarily targets the RPE. The exact mode of cell death induced by NaIO_3_, however, has not yet been fully elucidated [30]. Similar to H_2_O_2_, lower concentrations of NaIO_3_ have been reported to induce apoptosis, while higher concentrations are thought to induce necrosis [30,31]. Conversely, it has also been proposed that NaIO_3_ induces necroptosis [16,30,32], pyroptosis [33], or ferroptosis [34] in RPE cells. It is not unlikely that the pathway of cell death may be concentration dependent. In our cells, we found a threshold effect for NaIO_3_ between 10 and 20 mM in most (but not all) conditions tested, implying a change in cell death pathways to necrosis at 20 mM.

CoCl_2_ simulates hypoxia by activating HIF, inducing an increased production of ROS in the cells [24]. The time frame (24 h) and concentration (250 µM) determined as most suitable in this study are in line with previous publications using RPE cells [24]. Of note, the concentration is lower than what has been used for ARPE-19 cells [35,36,37].

Erastin, on the other hand, is inducing ferroptosis [38]. It basically induces oxidative stress via reducing the ability of the RPE cell to cope with it by reducing GPX4 expression and GSH levels [38,39]. In our model, a concentration of 25 µM erastin induced reproducible cell death close to 50%. The concentration was higher than needed for ARPE-19 cells [13], which again is in line with the higher resistance against oxidative stress found in primary RPE cells. Because of the high reproducibility and low standard deviation, we recommend 25 µM erastin (on uncoated wells and in 5% serum) for standard testing of oxidative stress.

It has to be mentioned, however, that the use of the stressor is also dependent on the objective of this study. Especially considering the testing of compounds against oxidative stress, the pathway the putative inhibitor is addressing needs to be considered when choosing the stressor. The different oxidative stressors tested in this study induce cell death via different mechanisms, using different pathways of inducing oxidative stress and executing cell death via different cell death pathways. If a compound acts as a scavenger, a direct oxidative stress such as H_2_O_2_ is advised; a compound that interferes with ferroptosis will be well suited with erastin, and a potential therapeutic interfering with HIF-mediated signaling will exert its effects best with CoCl_2_. Also, for these stressors, this study provides optimized protocols.

In addition, the protocols can and should be amended concerning the research objective. For oxidative stress, the use of coating is not needed if viability is the read-out. However, if gene expression is to be tested, PDL coating is recommended. We have previously shown that PDL coating confers the best differentiation in our primary cells [3]. As highly differentiated RPE cells are highly resistant to oxidative stress, we amended our culture accordingly, using 7-day-old cultures, cultivated without PDL, which are generally confluent but have not yet developed a fully differentiated RPE layer [3]. While this is appropriate for viability, in the case of proper gene expression, it is feasible that a more differentiated RPE is needed for reproducible gene expression changes, which can be achieved by PDL coating. Our cells are highly heterogeneous between cultures (as they are derived from a single eye each), which makes statistically significant changes in gene expression more difficult to achieve than in other models, such as cell lines or inbred strains. Similarly, while cytokine secretion is best assessed in the non-polar model where no coating is needed, for reproducible (and statistically significant) gene expression changes, the higher differentiation achieved in the polar model, coated with Lam [4], is recommended.

Also, we are aware of the limitations of the MTT assay used for studying cell toxic effects. Sometimes, stressors tend to increase the signal found in MTT. As MTT is measuring metabolic activity (as a correlate to viability), the apparent increase in viability reflects an increase in activity the sublethally stressed cells initiate to countermeasure the stress or an increased proliferation after stress insult [40].

In addition, we compared three inducers of inflammatory responses in the RPE, LPS, Poly I:C, and TNF, investigating IL-8, IL-6, and TNF secretion. Generally, the strongest response was found for LPS, which is in accordance with our previous studies on mixed RPE cultures [15]. We therefore recommend LPS (on uncoated wells in 5% serum) as a stressor for investigating pro-inflammatory activation of RPE cells. However, also Poly I:C will induce a reproducible secretion of cytokines, and the final choice will depend on the pathways of interest. LPS is an agonist of TLR-4 [41], while Poly I:C is activating TLR-3 [42], thereby facilitating different pathways for pro-inflammatory activation. Similar to oxidative stress, the final choice of the stressor has to be based on the objective to be studied. For example, many fucoidans interfere with TLR-3 but not TLR-4-mediated cytokine secretion [43].

Of note, in contrast to mixed RPE cultures, which often display cytokine secretion in their controls (especially seen for IL-8) [44], the single-eye primary RPE cells generally displayed little activation in their controls. As the mixed cultures contain RPE cells of several different individuals, it is feasible that allogenic stimuli induce a pro-inflammatory activation in these cells, which would render the single-eye RPE cell culture more suitable. A drawback, however, is the high standard deviation of the concentrations secreted, which is likely to be a result of the different genetic backgrounds of the cultures. However, as pointed out before, genetic heterogeneity makes this model biologically more relevant and can be met by a higher sample size.

Finally, our models and the protocols only target two pathways that are involved in AMD—oxidative stress and inflammation. AMD is a multifactorial disease, and additional pathways include angiogenesis, complement activation, lipid accumulation, and lipid peroxidation, amongst others [45]. Furthermore, our model features only RPE cells, and while the RPE is strongly involved in the development of AMD, the pathogenesis of AMD takes place in the interaction of the RPE with the photoreceptors above and the choroid beyond [46], with the potential involvement of microglia and macrophages [47,48]. Therefore, further development is needed to mimic further pathomechanisms, to combine different insults important for AMD development, and to develop an in vitro system combining several types of cells involved in AMD pathogenesis [2]. Primary porcine single-eye RPE cultures may serve as a starting point for these new models, combining economic feasibility with high biological relevance.

Taken together, we present optimized protocols for testing oxidative stress and inflammation in single-eye primary RPE cells, offering easy-to-handle, biologically relevant models.

## 4. Materials and Methods

### 4.1. Single-Eye Retinal Pigment Epithelium Preparation

Primary porcine RPE was prepared from pigs’ eyes (Deutsche Landrasse, Duroc, hybrid; mixed sex; half a year old) 4 to 5 h post mortem. The eyes are routinely removed before processing the pig further in food production. The use of these eyes in research was agreed upon by the animal welfare officer of the University of Kiel according to the German Animal Welfare Act (TierSchG, https://www.gesetze-im-internet.de/tierschg, accessed on 13 July 2025). It is not considered an animal experiment but a contribution to the reduction of those (3R Principle).

The preparation as single-eye cultures (cells from one eye into one well) was previously established and described with detailed protocol to follow [3]. In brief, adjacent tissue was removed and eyes treated with cold betaisodona (Mundipharma, Frankfurt am Main, Germany; #04923204), followed by washing in cold 0.9% NaCl (Fresenius Kabi, Bad Homburg, Germany; #04801702). Eyes were cut open and retinas removed in Dulbecco’s phosphate-buffered saline without Ca^2+^ and Mg^2+^ (DPBS, Pan-Biotech, Aidenbach, Germany; #P04-53500) plus 1% penicillin/streptomycin (Pe/St, Sigma-Aldrich, St. Louis, MO, USA; #P0781). Eyes were filled with 37 °C warm 0.25% trypsin (Pan-Biotech, Aidenbach, Germany; #P10-021100) and 0.25% trypsin/0.02% ethylenediaminetetraacetic acid (EDTA, Pan-Biotech, Aidenbach, Germany; #P10-020100) for 10 and 35 min, respectively. Cells were washed two times with 5 mL media consisting of Dulbecco’s Modified Eagle Medium with high glucose, L-glutamine, and phenol red (DMEM, Gibco™, Thermo Fisher Scientific, Waltham, MA, USA; #41965062), 1% non-essential amino acids (Pan-Biotech, Aidenbach, Germany; #P08-32100), 11 mM sodium pyruvate (Pan-Biotech, Aidenbach, Germany; #P04-43100), 1% Pe/St, and 10% fetal bovine serum (FBS Mexican origin, Gibco™, Thermo Fisher Scientific, Waltham, MA, USA; #10437028, Lot: 2405703RP). Cells were resuspended in 1 mL media and seeded into one well of a 12-well plate (Sarstedt, Nümbrecht, Germany; #83.3921) and kept at 37 °C and 5% CO_2_. For polar differentiation of the RPE (for RPE-Inf), cells were seeded on 0.4 µm transwell inserts (Sarstedt, Nümbrecht, Germany; #83.3931.041).

To assess cell number, 20 µL of cell suspension were applied to a acella counting chamber of the fluidlab R-300 cell counter device (anvajo Biotech GmbH, Dresden, Germany; # 1340184). After determining the optimal seeding cell number, cells were seeded with 100,000 cells/mL.

For serum testing, the serum content of the media was changed from 10% to 5% or 1% during the first media change, three days (d) after preparation. To check barrier properties, transepithelial resistance was measured with the EVOM3 Epithelial Volt/Ohm (TEER) Meter (World Precision Instruments Germany GmbH, Friedberg, Germany) prior to and post stimulation. Wells with TEER of at least 100 Ω·cm^2^ were used for stimulation.

### 4.2. Coating of Microtiter Plates or Transwell Inserts

Coating agents were used to coat individual wells of the 12-well plate or the integrated membrane of the transwells, depending on the model [3,4]. For polar RPE-Inf experiments, indicated transwells were coated with Lam (Sigma-Aldrich, Nümbrecht, Germany; #11243217001). For 12-well plates of RPE-Ox and RPE-Inf, PDL (Sigma-Aldrich, Nümbrecht, Germany; #P7886) was used for the indicated wells. Both coatings were applied as described in the corresponding manufacturer’s instructions with recommended doses (Lam—20 µg/mL or PDL—100 µg/mL or 10 µg/cm^2^) and well volumes (100 µL/cm^2^ well).

### 4.3. Stimulation Experiments

For RPE-Ox, cells were stimulated 5, 7, or 14 d after preparation on uncoated or PDL-coated 12-well plates with 1%, 5%, or 10% serum-containing media. Cells were stimulated with 250 µM H_2_O_2_ (Sigma-Aldrich; #H1009), 500 µM H_2_O_2_, 20 µM erastin (Cayman Chemical, Ann Arbor, MI, USA; #CAY17754), 25 µM erastin, 250 µM CoCl_2_ (Sigma-Aldrich, Nümbrecht, Germany; #232696), 500 µM CoCl_2_, 10 mM NaIO_3_ (Sigma-Aldrich, Nümbrecht, Germany; #S4007), or 20 mM NaIO_3_ for 4 or 24 h. After stimulation for the indicated period of time, cell viability was measured (Section 4.4) or RNA was isolated (Section 4.5).

For RPE-Inf, cells were stimulated at full confluence 14 to 18 d after preparation on uncoated or PDL-coated 12-well plates or uncoated or Lam-coated transwell inserts with 1%, 5%, or 10% serum-containing media. Cells were stimulated with 1 µg/mL LPS from *Escherichia coli* O55:B5 (Merck, Darmstadt, Germany; #L4524), 10 µg/mL Poly I:C (PIC, Tocris Bioscience, Bristol, UK; #4287/10), or 50 ng/mL recombinant human tumor necrosis factor alpha (TNF, R&D systems, Minneapolis, MN, USA; #210-TA-005/CF) for 1 or 3 d. Supernatants were collected (24 h collection time) and applied in ELISA, and cell viability was determined (Section 4.4), or RNA was isolated (Section 4.5). For transwell plates, TEER was measured before and after stimulation (Section 4.1).

### 4.4. Enzyme-Linked Immunosorbent Assay and Tetrazolium Assay

For RPE-Inf, supernatants of single-eye RPE were collected for 24 h after a cultivation time of 14–18 d and analyzed for interleukin 6 (IL-6), interleukin 8 (IL-8), and TNF secretion. They were applied in Porcine IL-6 DuoSet ELISA (R&D systems, Minneapolis, MN, USA; #DY686), Porcine IL-8/CXCL8 DuoSet ELISA (R&D systems, Minneapolis, MN, USA; #DY535), and Porcine TNF-alpha DuoSet ELISA (R&D systems, Minneapolis, MN, USA; #DY690B). All ELISAs were conducted as instructed by the manufacturer.

For RPE-Ox and RPE-Inf, viability of the cells was detected with tetrazolium bromide assay [3-(4,5-dimethylthiazol-2-yl)-2,5-diphenyltetrazolium bromide, MTT, Sigma-Aldrich, Nümbrecht, Germany; #M2128 [49]] after stimulation. Wells were washed with PBS, and 0.5 mg/mL MTT solution in DMEM without phenol red (Cytiva, Marlborough, MA, USA; #SH30284.01) was applied for 15 min at 37 °C. MTT was removed, and formazan crystals were dissolved in dimethyl sulfoxide (Carl Roth, Karlsruhe, Germany; #7029.1). Wells were measured at 550 nm absorption with Elx800 (BioTek, Bad Friedrichshall, Germany).

### 4.5. Quantitative Polymerase Chain Reaction

To assess gene expression, RNA was isolated after the indicated stimulation time using the NulceoSpin RNA Mini Kit, containing DNase to remove genomic DNA (Macherey-Nagel, Düren, Germany; #740955) as described by the manufacturer. RNA concentration and quality were assessed with NanoDrop™ One (Thermo Fisher Scientific, Waltham, MA, USA; #ND-ONE-W). cDNA was created with the High-Capacity cDNA Reverse Transcription Kit (Thermo Fisher Scientific, Waltham, MA, USA; #4368814) according to the instructions by Thermo Fisher Scientific. RT-qPCR was conducted with TaqMan™ gene expression assays (Table 5) with dye label 5(6)-carboxyfluorescein-minor groove binder (FAM-MGB) (Thermo Fisher Scientific, Waltham, MA, USA; #4351372) and TaqMan™ Fast Advanced Master Mix (Thermo Fisher Scientific, Waltham, MA, USA; #4444557) as described in the manual of the master mix. *GUSB* was used as an endogenous control. For samples of RPE-OX, RNA was collected after 7 or 14 d of cultivation using PDL or no coating, as well as 1%, 5%, or 10% serum, respectively. Gene targets were *CAT*, *GPX4*, *GSS*, *NFE2L2,* and *SOD1*. For samples of RPE-Inf, RNA was collected after 14–18 d of cultivation using PDL or no coating, as well as 5% or 10% serum. Gene targets were *CXCL8*, *IL1B*, *IL6*, and *NOS2*. For all data, relative gene expression according to the ΔΔCT method [50] was calculated with Thermo Fisher Connect (RQ module, relative quantification).

### 4.6. Statistical Analysis

All experiments were performed at least three times (the number of experiments, *n,* represents the number of individual eyes used in a given assay). Data managing, calculations, and diagrams were conducted with Microsoft Excel (Excel 2010, Microsoft, Redmond, WA, USA). The RT-qPCR data and statistics were evaluated online with Thermo Fisher Connect. Other statistics were assessed with GraphPad Prism 10 (version 10.6.0, GraphPad Software, Inc., San Diego, CA, USA). Data distribution was assessed with a Q-Q plot. Parametric data were evaluated with ANOVA (analysis of variance), followed by Student’s *t*-test. Concerning MTT data for cell viability, a version of Student’s *t*-test, namely the one-sample *t*-test, was conducted, which calculated statistical significance between the experimental data against the 100% untreated control value (as a hypothetical fixed value). Non-parametric data were evaluated with the Kruskal–Wallis test, followed by the Mann–Whitney test (unpaired) or the Wilcoxon test (paired). Significance was considered with *p*-values < 0.05.

## 5. Conclusions

The aim of this study was to establish standard operation protocols for single-eye porcine retinal pigment epithelium preparation regarding relevant models for oxidative stress and inflammation, relevant for age-related macular degeneration. Regarding the oxidative stress model, effective toxicity was determined with 25 µm erastin (seven days of cultivation, 24 h of stimulation) in 5% serum cultures. Regarding inflammation models, three days of 1 µg/mL LPS stimulation showed effective cytokine activation with 5% serum. In both models, uncoated 12-well plates should be used, unless gene expression is the objective. In this case, PDL should be used in oxidative stress assays and polar cultures with Lam coating in the inflammation model. With that, two specific best practice protocols for usage of porcine single-eye cultures in age-related macular degeneration research with optimized parameters were established.

## Figures and Tables

**Table 1:** Oxidative stress insult in single-eye cultures, gene expression. Primary porcine retinal pigment epithelium single-eye cultures were treated with 25 µM erastin, 500 µM CoCl_2_, or 10 mM NaIO_3_ for 24 h after a cultivation time of 7 d or 14 d on 12-well plates uncoated or coated with Poly-ᴅ-Lysine (PDL) using 10%, 5%, or 1% serum. RNA was isolated, and RT-qPCR was performed. *GUSB* was used as an endogenous control. Data were evaluated with Thermo Fisher Connect. * *p* < 0.05, ** *p* < 0.01, *** *p* < 0.001, *n* = 6. Rq = Relative Quantification.

Bio Group Name	Cultivation Time	Serum Content	Coating	Target Name	Rq	Rq Min	Rq Max	*p*-Value
10 mM NaIO_3_	7 d	1%	none	*CAT*	0.11	0.02	0.54	0.023 *
10 mM NaIO_3_	7 d	1%	none	*GPX4*	0.28	0.08	1.00	0.121
10 mM NaIO_3_	7 d	1%	none	*GSS*	0.39	0.11	1.35	0.137
10 mM NaIO_3_	7 d	1%	none	*NFE2L2*	0.11	0.00	3.17	0.177
10 mM NaIO_3_	7 d	1%	none	*SOD1*	0.70	0.21	2.41	0.610
10 mM NaIO_3_	7 d	1%	PDL	*CAT*	0.12	0.07	0.21	<0.001 ***
10 mM NaIO_3_	7 d	1%	PDL	*GPX4*	0.23	0.08	0.61	0.045 *
10 mM NaIO_3_	7 d	1%	PDL	*GSS*	0.45	0.29	0.72	0.226
10 mM NaIO_3_	7 d	1%	PDL	*NFE2L2*	0.22	0.11	0.45	0.021 *
10 mM NaIO_3_	7 d	1%	PDL	*SOD1*	0.37	0.29	0.48	0.088
25 µM erastin	7 d	5%	none	*CAT*	1.65	0.33	8.32	0.504
500 µM CoCl_2_	7 d	5%	none	*CAT*	0.38	0.11	1.31	0.132
25 µM erastin	7 d	5%	none	*GPX4*	1.03	0.23	4.67	0.976
500 µM CoCl_2_	7 d	5%	none	*GPX4*	0.87	0.26	2.98	0.877
25 µM erastin	7 d	5%	none	*GSS*	4.93	1.54	15.74	0.075
500 µM CoCl_2_	7 d	5%	none	*GSS*	0.90	0.34	2.36	0.889
25 µM erastin	7 d	5%	none	*NFE2L2*	5.14	0.82	32.20	0.085
500 µM CoCl_2_	7 d	5%	none	*NFE2L2*	1.87	0.74	4.72	0.223
25 µM erastin	7 d	5%	none	*SOD1*	0.95	0.18	4.96	0.945
500 µM CoCl_2_	7 d	5%	none	*SOD1*	0.85	0.25	2.87	0.784
25 µM erastin	7 d	5%	PDL	*CAT*	0.92	0.37	2.30	0.865
500 µM CoCl_2_	7 d	5%	PDL	*CAT*	0.36	0.12	1.06	0.063
25 µM erastin	7 d	5%	PDL	*GPX4*	0.68	0.25	1.83	0.568
500 µM CoCl_2_	7 d	5%	PDL	*GPX4*	0.96	0.30	3.10	0.948
25 µM erastin	7 d	5%	PDL	*GSS*	1.12	0.42	2.99	0.876
500 µM CoCl_2_	7 d	5%	PDL	*GSS*	0.62	0.09	4.37	0.605
25 µM erastin	7 d	5%	PDL	*NFE2L2*	1.35	0.36	5.07	0.697
500 µM CoCl_2_	7 d	5%	PDL	*NFE2L2*	0.49	0.19	1.25	0.270
25 µM erastin	7 d	5%	PDL	*SOD1*	3.04	1.73	5.34	0.011 *
500 µM CoCl_2_	7 d	5%	PDL	*SOD1*	1.86	0.59	5.86	0.230
25 µM erastin	7 d	10%	none	*CAT*	1.90	1.05	3.42	0.427
500 µM CoCl_2_	7 d	10%	none	*CAT*	1.46	0.22	9.61	0.717
25 µM erastin	7 d	10%	none	*GPX4*	0.45	0.29	0.70	0.078
500 µM CoCl_2_	7 d	10%	none	*GPX4*	0.54	0.06	4.57	0.502
25 µM erastin	7 d	10%	none	*GSS*	1.04	0.29	3.78	0.963
500 µM CoCl_2_	7 d	10%	none	*GSS*	1.10	0.03	36.92	0.949
25 µM erastin	7 d	10%	none	*NFE2L2*	1.68	0.51	5.53	0.345
500 µM CoCl_2_	7 d	10%	none	*NFE2L2*	1.58	0.25	9.93	0.538
25 µM erastin	7 d	10%	none	*SOD1*	0.79	0.37	1.70	0.530
500 µM CoCl_2_	7 d	10%	none	*SOD1*	1.43	0.19	10.69	0.662
25 µM erastin	7 d	10%	PDL	*CAT*	1.44	0.53	3.94	0.422
500 µM CoCl_2_	7 d	10%	PDL	*CAT*	0.39	0.18	0.84	0.088
25 µM erastin	7 d	10%	PDL	*GPX4*	0.44	0.12	1.66	0.194
500 µM CoCl_2_	7 d	10%	PDL	*GPX4*	0.25	0.04	1.74	0.250
25 µM erastin	7 d	10%	PDL	*GSS*	2.31	1.03	5.19	0.056
500 µM CoCl_2_	7 d	10%	PDL	*GSS*	0.70	0.40	1.22	0.321
25 µM erastin	7 d	10%	PDL	*NFE2L2*	2.84	1.31	6.17	0.036 *
500 µM CoCl_2_	7 d	10%	PDL	*NFE2L2*	1.32	0.67	2.63	0.554
25 µM erastin	7 d	10%	PDL	*SOD1*	2.45	1.38	4.34	0.126
500 µM CoCl_2_	7 d	10%	PDL	*SOD1*	1.00	0.55	1.81	0.996
10 mM NaIO_3_	14 d	10%	none	*CAT*	0.21	0.07	0.63	0.024 *
10 mM NaIO_3_	14 d	10%	none	*GPX4*	0.63	0.21	1.84	0.528
10 mM NaIO_3_	14 d	10%	none	*GSS*	0.99	0.36	2.71	0.993
10 mM NaIO_3_	14 d	10%	none	*NFE2L2*	1.21	0.50	2.91	0.763
10 mM NaIO_3_	14 d	10%	none	*SOD1*	1.14	0.39	3.28	0.842
10 mM NaIO_3_	14 d	10%	PDL	*CAT*	0.10	0.05	0.22	0.003 **
10 mM NaIO_3_	14 d	10%	PDL	*GPX4*	0.05	0.01	0.37	0.015 *
10 mM NaIO_3_	14 d	10%	PDL	*GSS*	0.64	0.08	5.30	0.728
10 mM NaIO_3_	14 d	10%	PDL	*NFE2L2*	0.23	0.10	0.53	0.074
10 mM NaIO_3_	14 d	10%	PDL	*SOD1*	0.31	0.07	1.40	0.145

**Table 2:** Pro-inflammatorily activated single-eye RPE cultures and their gene expression. Primary porcine retinal pigment epithelium single-eye cultures were treated with 1 µg/mL lipopolysaccharide (LPS) or 10 µg/mL polyinosinic/polycytidylic acid (Poly I:C, PIC) for 3 d on 12-well plates uncoated or coated with Poly-ᴅ-Lysine (PDL) using 10% or 5% serum. RNA was isolated, and RT-qPCR was performed. *GUSB* was used as an endogenous control. Data were evaluated with Thermo Fisher Connect. * *p* < 0.05, *n* = 6. Rq = Relative Quantification.

Bio Group Name	Serum Content	Coating	Target Name	Rq	Rq Min	Rq Max	*p*-Value
1 µg/mL LPS	5%	none	*IL1B*	2.49	1.16	5.36	0.158
10 µg/mL PIC	5%	none	*IL1B*	0.32	0.08	1.28	0.164
1 µg/mL LPS	5%	none	*IL6*	0.68	0.46	1.00	0.248
10 µg/mL PIC	5%	none	*IL6*	0.44	0.25	0.80	0.048 *
1 µg/mL LPS	5%	none	*CXCL8*	1.33	0.48	3.65	0.599
10 µg/mL PIC	5%	none	*CXCL8*	0.40	0.26	0.61	0.036 *
1 µg/mL LPS	5%	none	*NOS2*	0.55	0.28	1.04	0.148
10 µg/mL PIC	5%	none	*NOS2*	0.72	0.38	1.34	0.400
1 µg/mL LPS	10%	none	*IL1B*	8.04	3.35	19.29	0.035 *
10 µg/mL PIC	10%	none	*IL1B*	2.09	0.87	5.01	0.392
1 µg/mL LPS	10%	none	*IL6*	1.85	0.87	3.94	0.208
10 µg/mL PIC	10%	none	*IL6*	0.90	0.40	2.02	0.829
1 µg/mL LPS	10%	none	*CXCL8*	2.07	1.29	3.34	0.060
10 µg/mL PIC	10%	none	*CXCL8*	1.15	0.48	2.80	0.760
1 µg/mL LPS	10%	none	*NOS2*	1.10	0.81	1.48	0.706
10 µg/mL PIC	10%	none	*NOS2*	0.69	0.44	1.09	0.201
1 µg/mL LPS	5%	PDL	*IL1B*	3.00	1.48	6.11	0.230
10 µg/mL PIC	5%	PDL	*IL1B*	0.70	0.19	2.62	0.716
1 µg/mL LPS	5%	PDL	*IL6*	1.78	0.86	3.68	0.343
10 µg/mL PIC	5%	PDL	*IL6*	1.10	0.81	1.50	0.853
1 µg/mL LPS	5%	PDL	*CXCL8*	2.19	0.60	8.05	0.352
10 µg/mL PIC	5%	PDL	*CXCL8*	1.09	0.50	2.40	0.901
1 µg/mL LPS	5%	PDL	*NOS2*	0.64	0.24	1.71	0.341
10 µg/mL PIC	5%	PDL	*NOS2*	0.90	0.65	1.25	0.662

**Table 3:** Pro-inflammatorily activated polar single-eye RPE cultures, gene expression. Primary porcine retinal pigment epithelium single-eye cultures were treated with 1 µg/mL lipopolysaccharide (LPS) or 10 µg/mL polyinosinic/polycytidylic acid (Poly I:C, PIC) for 3 d on transwell inserts not coated or coated with laminin (Lam) using 10% or 5% serum. RNA was isolated, and RT-qPCR was performed. *GUSB* was used as an endogenous control. Data were evaluated with Thermo Fisher Connect. * *p* < 0.05, ** *p* < 0.01, *n* = 6. Rq = Relative Quantification.

Bio Group Name	Serum Content	Coating	Target Name	Rq	Rq Min	Rq Max	*p*-Value
1 µg/mL LPS	5%	none	*IL1B*	2.53	1.19	5.36	0.242
10 µg/mL PIC	5%	none	*IL1B*	0.23	0.02	2.78	0.258
1 µg/mL LPS	5%	none	*IL6*	1.14	0.65	1.98	0.718
10 µg/mL PIC	5%	none	*IL6*	0.33	0.13	0.85	0.042 *
1 µg/mL LPS	5%	none	*CXCL8*	1.60	0.86	2.99	0.204
10 µg/mL PIC	5%	none	*CXCL8*	0.78	0.55	1.10	0.388
1 µg/mL LPS	5%	none	*NOS2*	1.84	1.29	2.64	0.122
10 µg/mL PIC	5%	none	*NOS2*	0.21	0.05	0.81	0.040 *
1 µg/mL LPS	10%	none	*IL1B*	0.77	0.27	2.19	0.636
10 µg/mL PIC	10%	none	*IL1B*	0.41	0.18	0.96	0.088
1 µg/mL LPS	10%	none	*IL6*	0.87	0.65	1.17	0.548
10 µg/mL PIC	10%	none	*IL6*	0.68	0.32	1.44	0.318
1 µg/mL LPS	10%	none	*CXCL8*	1.39	0.46	4.25	0.545
10 µg/mL PIC	10%	none	*CXCL8*	0.94	0.44	2.02	0.888
1 µg/mL LPS	10%	none	*NOS2*	1.11	0.69	1.79	0.662
10 µg/mL PIC	10%	none	*NOS2*	0.95	0.63	1.42	0.792
1 µg/mL LPS	5%	Lam	*IL1B*	1.20	0.35	4.05	0.758
10 µg/mL PIC	5%	Lam	*IL1B*	0.30	0.06	1.62	0.151
1 µg/mL LPS	5%	Lam	*IL6*	1.13	0.68	1.88	0.659
10 µg/mL PIC	5%	Lam	*IL6*	0.75	0.44	1.25	0.313
1 µg/mL LPS	5%	Lam	*CXCL8*	2.84	1.79	4.52	0.006 **
10 µg/mL PIC	5%	Lam	*CXCL8*	0.58	0.25	1.36	0.223
1 µg/mL LPS	5%	Lam	*NOS2*	0.93	0.34	2.59	0.882
10 µg/mL PIC	5%	Lam	*NOS2*	0.16	0.04	0.65	0.022 *

**Table 4:** Pre-stimulation barrier. Mean and standard deviation (STD) of all single-eye culture transwell barrier measurements (TEER) sorted by serum content and coating. *n* = 51–98.

	1% Serum	5% Serum	10% Serum
Mean	199.31 Ω·cm^2^	247.01 Ω·cm^2^	316.74 Ω·cm^2^
STD	137.36 Ω·cm^2^	181.18 Ω·cm^2^	251.39 Ω·cm^2^
	+ Lam	+ Lam	+ Lam
Mean	193.18 Ω·cm^2^	248.81 Ω·cm^2^	212.00 Ω·cm^2^
STD	126.24 Ω·cm^2^	205.89 Ω·cm^2^	158.88 Ω·cm^2^

**Table 5:** Gene targets for RPE-OX and RPE-Inf. In this table, all gene targets and gene expression IDs are depicted. Also, the name of the encoded gene product can be seen. The underlined gene was used as an endogenous control.

Gene	Product	Assay ID
*CAT*	Catalase	Ss04323025_m1
*CXCL8*	Interleukin 8	Ss03392437_m1
*GPX4*	Glutathione Peroxidase 4	Ss03384646_u1
*GSS*	Glutathione Synthetase	Ss04328106_m1
*GUSB*	Glucuronidase Beta	Ss03387751_u1
*IL1B*	Interleukin 1 Beta	Ss03393804_m1
*IL6*	Interleukin 6	Ss07308316_g1
*NFE2L2*	Nuclear Factor Erythroid 2-Related Factor 2 (Nrf2)	Ss06886076_m1
*NOS2*	Nitric Oxide Synthase 2	Ss03374608_u1
*SOD1*	Superoxide Dismutase 1	Ss03375614_u1

## Data Availability

Data can be provided upon request.
